# Chaperonin containing TCP-1 (CCT/TRiC) is a novel therapeutic and diagnostic target for neuroblastoma

**DOI:** 10.3389/fonc.2022.975088

**Published:** 2022-09-15

**Authors:** Amanda Cox, Daniel Nierenberg, Oscar Camargo, Eunkyung Lee, Amr S. Khaled, Joseph Mazar, Rebecca J. Boohaker, Tamarah J. Westmoreland, Annette R. Khaled

**Affiliations:** ^1^ Burnett School of Biomedical Science, College of Medicine, University of Central Florida, Orlando, FL, United States; ^2^ College of Health Professions and Sciences, University of Central Florida, Orlando, FL, United States; ^3^ Pathology and Laboratory Medicine, Orlando VA Medical Center, Orlando, FL, United States; ^4^ Department of Oncology, Southern Research Institute, Nemours Children’s Hospital, Orlando, FL, United States; ^5^ Department of Biomedical Research, Nemours Children’s Hospital, Southern Research, Birmingham, AL, United States

**Keywords:** cancer, pediatric, peptide, circulating tumor cells, chaperone, protein-folding, liquid biopsy, nanoparticles

## Abstract

Chaperonin containing TCP1 (CCT/TRiC) is a multi-subunit protein folding complex that enables the cancer phenotype to emerge from the mutational landscape that drives oncogenesis. We and others linked increased expression of CCT subunits to advanced tumor stage and invasiveness that inversely correlates with cancer patient outcomes. In this study, we examined the expression of the second CCT subunit, CCT2, using genomic databases of adult and pediatric tumors and normal tissues, and found that it was highly expressed in pediatric cancers, showing a significant difference compared to normal tissues. Histologic staining confirmed that CCT subunits are highly expressed in tumor tissues, which was exemplified in neuroblastoma. Using two neuroblastoma cells, MYCN-amplified, IMR-32 cells, and non-amplified, SK-N-AS cells, we assessed baseline levels for CCT subunits and found expressions comparable to the highly invasive triple-negative breast cancer (TNBC) cell line, MDA-MB-231. Exogenous expression of CCT2 in both SK-N-AS and IMR-32 cells resulted in morphological changes, such as larger cell size and increased adherence, with significant increases in the CCT substrates, actin, and tubulin, as well as increased migration. Depletion of CCT2 reversed these effects and reduced cell viability. We evaluated CCT as a therapeutic target in IMR-32 cells by testing a novel peptide CCT inhibitor, CT20p. Treatment with CT20p induced cell death in these neuroblastoma cells. The use of CCT2 as a biological indicator for detection of neuroblastoma cells shed in blood was examined by spiking IMR-32 cells into human blood and using an anti-CCT2 antibody for the identification of spiked cancer cells with the CellSearch system. Results showed that using CCT2 for the detection of neuroblastoma cells in blood was more effective than the conventional approach of using epithelial markers like cytokeratins. CCT2 plays an essential role in promoting the invasive capacity of neuroblastoma cells and thus offers the potential to act as a molecular target in the development of novel therapeutics and diagnostics for pediatric cancers.

## Introduction

Neuroblastoma is an extracranial solid tumor of childhood and is the most common cancer diagnosed in children under one year of age (1). Neuroblastoma is highly heterogeneous and forms from immature nerve tissue along the peripheral sympathetic nervous system. The most common location for neuroblastoma is in the nerve tissue of the adrenal glands located above the kidneys (1). Clinical behavior ranges from tumors that spontaneously regress to aggressive metastatic disease that disseminates and causes patient death. With only 1-2% of neuroblastoma cases having genetic or familial links (2), many of the underlying molecular factors responsible for this diverse clinical picture remain unknown. Patients are generally diagnosed after presenting with symptoms such as an abnormal mass in the abdomen, high blood pressure, enlarged lymph nodes, and bone pain. Once diagnosed with neuroblastoma, classification of the patient’s risk for regression or metastasis informs treatment approaches. Methods for classification of risk status include clinical and biological factors such as age, tumor histology, deoxyribonucleic acid (DNA) ploidy, *MYCN* gene amplification, and chromosome changes in 1p, 11q, and 17q (1–3), **Table 1**. Low-risk patients have >85% 5-year event-free survival, while high-risk patients have <50% 5-year event-free survival (4). Treatments for the high-risk disease usually involve combinations of surgery, chemo- and radiation therapies, and often necessitate the use of drugs made for adult cancers which can have harmful side effects on children, especially for the high-risk neuroblastoma groups that require multimodality therapies or those with cancer recurrence. Children who survive neuroblastoma after intensive treatments are at a heightened risk for long-term morbidity and mortality concerns. Consequently, the cost of treatment associated with neuroblastoma is often significant in terms of physical side effects, long-term impairment, and emotional or social ramifications. Addressing this unmet medical need obligates the discovery of viable molecular targets to develop novel therapeutics and diagnostics that could improve patient outcomes.

**Table 1:** Neuroblastoma stages and risk classification factors.

Stage	Description
**1/L1**	Localized, no lymph node involvement
**2-3/L2**	Loco-regional, some lymph node involvement
**4/M**	Metastatic
**4S/MS**	Metastatic but <18 months old and limited to bone marrow, skin and/or liver
**Risk Type**	**Example Factors**
**Low Risk**	MYCN-down regulated; Hyperdiploidy, <18 months old
**High Risk**	MYCN amplified, Hypodiploidy, >18 months old; chromosome 1q deletion

Research from our lab and others demonstrated that targeting protein folding for cancer therapy could significantly reduce tumor growth (5–8). The term “chaperone addiction” was coined to describe the dependency of cancerous cells on the cellular protein folding machinery (9, 10). Molecular chaperones, like heat shock proteins (HSPs) or multi-subunit chaperonins, are key components of the processes that govern the cellular proteome that could be targeted therapeutically. While inhibitors of HSPs, like HSP90, have undergone decades of pre-clinical and clinical testing, none have shown the satisfactory efficacy needed to be approved for patient use (11). Part of the reasons for this failure is clarified by the fact that the past HSP90 inhibitors may cause dose-limiting toxicities and also elicit a cytoprotective heat shock response (12). Instead, an alternative approach to inhibit protein folding in cancer cells may be to target chaperonins.

Mammalian cells contain an evolutionarily conserved type II chaperonin called chaperonin containing tailless complex polypeptide 1 (CCT) or tailless complex polypeptide 1 ring complex (TRiC). The CCT complex is composed of eight subunits [CCT1-8 (yeast) or CCTα-θ (mammals)] and folds substrates needed for cell invasion and proliferation, such as actin, tubulin, and cell division cycle protein 20 homolog (cdc20), as well as oncoproteins like signal transducer and activator of transcription 3 (STAT3), Kirsten rat sarcoma viral oncogene homolog (KRAS), and Myelocytomatosis (MYC) (8, 13–17). CCT-interacting proteins are found in the ten major signaling pathways aberrantly expressed in diverse cancers (6, 18). As a result, CCT subunits are often upregulated in breast, prostate, gastric, colon, lung, and other cancers (19–27). In particular, expression of the second CCT subunit, CCT2, correlated with increased tumor stage and aggression while inversely correlating with cancer patient outcomes (7, 19–22, 26). Overexpression of CCT2 increased the proliferation and invasiveness of breast epithelial cells (7) and promoted spheroid and anchorage-independent growth of luminal A breast cancer cells, correlating with increased *MYC* and *CCND1* expression (7, 8). Depletion of CCT2 prevented the growth of TNBC in syngeneic mice (7). Such data suggest that CCT2 may have a unique and essential activity in the chaperonin complex. CCT2 is postulated to be the first subunit to load onto a CCT5 homo-oligomer “template” that initiates assembly of the CCT complex (28), has a long half-life (~8 hrs) (29), and has one of the highest affinities for ATP (30). Cryogenic electron microscopy (cryo-EM) structures revealed that the CCT2 homodimer forms a Z shape in the complex that supports the allosteric cooperativity (30). Relevant to cancer in humans, *CCT2* is located in a genetic amplification hotspot on chromosome 12q15 with other oncogenes like *YEATS4, FRS2*, and *MDM2* (8). Taken together, these findings indicate that CCT2 could be a readout for increased activity of the CCT complex, as our lab showed with breast cancer models (7), and has practical applications as a diagnostic indicator and therapeutic target in cancer.

In this study, we investigated the expression and activity of CCT in pediatric cancers. We rationalized that much like in adult cancers, CCT could function as an enabler of oncogenesis in pediatric cancers, providing key protein substrates for the survival, growth, and dissemination of malignant cells. Bioinformatic and histologic data revealed increased expression of CCT2 RNA and protein across multiple pediatric cancers, with some of the highest expression shown in neuroblastoma. Performing exogenous expression and depletion experiments with select neuroblastoma cell lines revealed that the CCT2 subunit is essential for the CCT complex to manifest the cancer phenotype of increased migration through its protein folding activity on cytoskeletal elements. Direct inhibition of CCT2, either using RNA interference or a CCT inhibitor developed by our lab, CT20p, was cytotoxic to neuroblastoma cells. The intracellular detection of CCT2 could also be used to identify neuroblastoma cells spiked into blood, suggesting a possible application as a diagnostic for the detection and enumeration of circulating tumor cells (CTCs) shed from pediatric tumors.

## Materials and methods

### Bioinformatic analysis

Data was collected from the University of California Santa Cruz (UCSC) Xena at xena.ucsc.edu using the combined cohort of “The Cancer Genome Atlas (TCGA), Therapeutically Applicable Research to Generate Effective Treatments (TARGET), and Genotype Tissue Expression (GTEx) samples” dataset (31). Expression for the *CCT2* gene was compared in a combined cohort of GTEx, TCGA, and TARGET samples (n= 19,131). Analysis, with specifically the TARGET dataset was completed for all eight CCT subunits. Samples were narrowed down by sample type to include only “Solid Tissue Normal” and “Primary Solid Tumor” (n=297). The Pediatric Brain Tumor Atlas: CBTTC cohort (n=961) in Kid’s First Xena Hub (https://kidsfirst.xenahubs.net) and the TARGET Pan-Cancer cohort (n=734) were analyzed separately for *CCT2* expression across the different cancer types. The TARGET Pan-Cancer cohort was restricted to include only “Solid Tissue Normal” and “Primary Solid Tumor” and was analyzed for nine different gene expressions: *CCT2, MYC, MYCN, CDK2, CDK4, CCND1, CCNE1, YAP1*, and *RB1* (n=297). Welch’s t-tests were used to compare the difference in gene expression between the two sample types.

### Immunohistochemistry (IHC) staining of tumor tissues for CCT2

Slides with formalin-fixed paraffin-embedded (FFPE) tissues were received from US Biomax: PC701 and NB642c. Slides were processed by standard IHC methods for CCT2 (26). Anti-CCTβ antibody [amino acids 277 and 473 of Human TCP1 beta] (LifeSpan Biosciences) was used. Slides were stained and scored for the CCT2 stain by an independent and identification-blinded pathologist, as described previously (21, 32). Digital images were acquired at 40X magnification using the BZ-X800 Keyence.

### Cell culture media and growth conditions

IMR-32 cells (ATCC^®^ CCL-12™) were cultured in Eagle’s Minimal Essential Medium (EMEM) (Corning) and supplemented with 10% fetal bovine serum (FBS) (Gemini), 1% Penicillin-Streptomycin (P/S) (Corning), and L-Glutamine. SK-N-AS cells (ATCC^®^ CRL-2137™) were cultured in Dulbecco’s Modified Eagle’s Medium (DMEM) (Corning) and supplemented with 10% FBS (Gemini), 1X Non-Essential Amino Acids, 1% (P/S) (Corning), and L-Glutamine.

### Exogenous expression and depletion of CCT2

IMR-32 and SK-N-AS cells were transfected with a lentiviral plasmid to express CCT2 with a FLAG tag (DYKDDDDK), and cell lines were selected for stable expression of plasmids as previously described (7); hereafter, referred to as IMR32-CCT2 or SKNAS-CCT2 cells. For maintenance of these cell lines, 0.5 μg/mL puromycin dihydrochloride (ThermoFisher) was added to culture media (above) for SK-N-AS cells and 1.0 μg/mL was used for IMR-32 cells. For SK-N-AS cells, inducible depletion of CCT2 was achieved by stable transduction with a SMARTvector doxycycline-inducible lentiviral shRNA-CCT2 as described previously (Dharmacon; clone Id: V3IHSMCG_9075131) (7). In these cells, CCT2 depletion was induced by the addition of 1 μg/mL of doxycycline (doxy). To assess the effects of CCT2 depletion on viability, transient transduction using the lentiviral CCT2-shRNA vector was performed (without puromycin selection), and viability was assessed with a 3-(4,5-dimethylthiazol-2-yl)-2,5-diphenyl-2H-tetrazolium bromide (MTT) assay that was completed after 96 hours (hrs) of transduction using Thiazolyl Blue Tetrazolium Bromide (Sigma Aldrich) according to the manufacturer’s protocol.

### Small interfering RNA (siRNA) treatment for IMR32 cells

IMR-32 cells were treated with ON-TARGETplus Human CCT2 siRNA (Dharmacon) which is a pool of four CCT2-targeting siRNAs using the DharmaFECT transfection reagents-siRNA transfection protocol (Horizon). The transfection reagent was used at 0.05 μL per 100 μL media. siRNAs were used at 5 μM. Cells were plated at a density of 4 x 10^4^ cells or 4 x 10^5^ cells. RNA was collected for analysis between 24-48 hrs post-treatment. The ATP assay, CellTiter-Glo 2.0 Assay (Promega), was used as a cell viability indicator and was measured between 72-96 hrs. post-treatment according to the manufacturer’s protocol.

### Quantification of real-time polymerase chain reaction (RT-qPCR)

RNA was isolated from cells using TRIzolTM (Invitrogen) following the manufacturer’s standard protocol for RNA extraction. RNA was quantified using a Nanodrop 8000 (ThermoFisher). Complementary DNA (cDNA) was synthesized using the iScriptTM cDNA Synthesis Kit (Bio-Rad) following the manufacturer’s protocol. The cDNA was diluted 1:5 and then mixed with a Fast SYBR™ Green Master Mix (Applied Biosystems) according to the manufacturer’s recommendations and amplified using a QuantStudio Real-Time PCR system (ThermoFisher Scientific) for 40 cycles at 95°C for 3 sec, and 62°C for 30 sec. Primers were designed using the National Center for Biotechnology Information (NCBI) nucleotide database, **Table S1**.

### Immunoblots

Cell lysis, sodium dodecyl sulfate polyacrylamide gel electrophoresis (SDS-PAGE), total protein measurements, gel visualization, and gel band quantification was performed as previously described (26). Total protein was determined using the Pierce BCA Protein Assay Kit (ThermoFisher Scientific) following the manufacturer’s protocol. Primary antibodies included: anti-CCT2 antibody (ab109184) (Abcam) which targets the N-terminal amino acids 1-100; CCT3 (MA5-27872) (Invitrogen); anti-CCT2 antibody (Millipore) which targets C-terminal amino acids; anti-FLAG antibody (ab1162, Abcam), anti-TCP1 delta antibody [EPR8495(B)] (ab129072, Abcam), and anti-TCP1 epsilon/CCT5 antibody [EPR7562] (ab129016, Abcam). Secondary antibodies included IRDye 800CW Goat anti-Mouse IgG and IRDye 680CW Goat anti-Rabbit IgG (LI-COR). Images were collected with a LiCor Odyssey Infrared Imaging System (LI-COR) and processed using ImageStudio software (LI-COR).

### Migration assay

Cells were cultured at a seed density of 50,000 cells (SK-N-AS) or 60,000 cells (IMR-32) per well in a 96-well plate overnight. The Scratch Wound Assay (Sartorius) was performed as described by the manufacturer and cells were washed and processed according to the recommended manufacturer’s protocol. Images were collected at hourly intervals for 24-hrs using the Incucyte S3 Live Cell Analysis System and analyzed using Incucyte 2021 A software.

### Cytoskeletal and nuclei staining of cells

To evaluate cytoskeletal elements in CCT2-FLAG expressing or control IMR-32 or SK-N-AS cells, cells were seeded between 10,000 to 30,000 cells per well in a 96-well plate or 300,000 cells per 6-well plate onto coverslips (Fisher Scientific). Cells were fixed, permeabilized, and stained for actin with phalloidin as described previously (8, 13–17). Staining for tubulin followed the same protocol except fixation was achieved with ice-cold methanol instead of buffered formalin. Probes used were: ActinRed 555 ReadyProbes Reagent (ThermoFisher), which stains F-actin, and anti-α tubulin antibody (DM1A) (Santa Cruz Biotechnology) with the secondary antibody, Alexa Fluor 568-conjugated goat anti-mouse IgG antibody (ThermoFisher). Cells on culture plates were visualized using the ImageXpress^®^ Pico (Molecular Devices, San Jose, CA, USA), at 20x magnification. Analysis was performed using the “Cell Count” function in the MD.CellReporterXpress (Molecular Devices) software. 4′,6-diamidino-2-phenylindole (DAPI) was used to track cell count and the TRITC channel was used for positive “marker” identification of actin or tubulin staining. Average positive cell area was used for calculations comparing parental and overexpressing cell lines. Images from fixed cells on microscope slide coverslips were also acquired using a Zeiss LSM 710 laser scanning confocal microscope (Carl Zeiss AG, Oberkochen, Germany) at 100x magnification.

### Production of recombinant CCT2 proteins and pull-down assay

Purified full-length HIS-CCT2 was generated from the codon-optimized cDNA sequence for CCT2 (**Table S2**). Based on this sequence, three constructs were expressed and purified for functional assays. The full-length was expressed in both BL21 (DE3) bacteria and Rosetta E. coli. The cells were pelleted and frozen at -80°C overnight. Upon thaw, cells were resuspended in a lysis buffer comprised of 20mM Tris, 500mM NaCl, 20mM imidazole, and 0.5mM TCEP. In addition, 1 mg/ml lysozyme and 1 tablet of SIGMAFAST were also added to the suspension. The suspension was sonicated and centrifuged at 15,000 rpm for 1-hr. The resultant lysate was mixed with approximately 1 mL of Ni-NTA beads and set to shake for 2-hrs. The beads were spun down to remove unbound material, then loaded into a gravity column. The beads were washed with 10 mL lysis buffer, followed by 10 mL of 40 mM imidazole, then 10 mL of 60 mM imidazole. The protein was eluted off the column with 400 mM imidazole. The affinity-purified protein was then loaded onto a size exclusion chromatography (SEC) column (HILoad 16 600 S200) and eluted off the column with elution buffer (20 mM Tris, 150 mM NaCl, 1 mM TCEP). The protein was purified as a single peak (**Figure S1A**). The yield was between 5 and 7 mg/mL respectively, approximately 18 mg/L. This full-length protein was then validated by western blot and pull-down to ensure that the protein was the target protein and that it was properly folded.

Truncations of CCT2 were also generated. We identified two truncation mutants: CCT2(150-407) and CCT2(17-526). These codon-optimized sequences were cloned into a pGEX-4T-3 vector (GE Healthcare) digested with BamHI and XhoI. Additionally, we replaced the HIS tag with a GST tag and replaced the thrombin cleavage site with a TEV protease cleavage site. These proteins were purified by GST-affinity column purification followed by SEC as described above. These truncations were also assessed for CT20p binding by pull-down assay, performed as described in our previous publication (21). Briefly, biotinylated-CT20p was commercially synthesized (BioPeptides Inc). For pull-downs, 0.1–50 nmol biotin-CT20p or biotin alone was mixed with recombinant CCT2 proteins (0.1 nmol), made above, in 20 mM Tris-HCl buffer and incubated for 3-hrs at room temperature, followed by overnight incubation at 4°C with streptavidin-agarose beads. Beads were washed and centrifuged, then prepared for SDS-PAGE analysis and immunoblot for CCT2 as described above.

### Nanoparticle uptake and CT20p treatment

For CT20p-based treatments, hyperbranched polyester nanoparticles (NP) were utilized. NP were synthesized and encapsulated with cargo as described previously (33). NP were loaded with either 1,1’-Dioctadecyl-3,3,3’,3’-Tetramethylindocarbocyanine Perchlorate (DiI dye) (ThermoFisher) or CT20p (Acetyl – VTIFVAGVLTASLTIWKKMG – Amine) peptide (Biopeptide Inc) as previously described (33, 34). DiI-loaded NP (0.02-0.1 mg) was used to assess uptake by IMR-32 cells and to detect any non-specific toxicity associated with NP carrier. For evaluating the toxicity of CT20p-NP, 0.01 mg or 0.02 mg of CT20p-loaded NP were used for treating IMR-32 cells for 24-hrs. PBS-treated cells served as negative controls. NP-treated IMR-32 cells were imaged with the Cytation 5 Cell Imaging Multi-Mode Reader (BioTek). DiI-NP uptake was visualized using a 531 nm excitation and 593 nm emission filter. Cell morphology was visualized with a brightfield channel. Images were taken at 10X magnification. Individual cell area counts before and after CT20p-NP treatment was assessed using Gen5 software (BioTek, Winooski, VT, USA).

### Detection of IMR-32 cells in blood

The manual staining protocol for cancer cells in buffer for antibody optimization was adapted from Lowes et al. (35). IMR-32 cells in 5% FBS/PBS were stained according to the protocol previously described (32), with the following modification: IMR-32 cells were washed twice with dilution buffer post-antibody staining. After staining, cells were re-suspended in dilution buffer and transferred into CellSearch MagNest cartridges and run in the CellSearch Analyzer II, and evaluated for CCT2-positive cells as described previously (32). For experiments with IMR-32 cells spiked into blood, 2 x 10^5^ IMR-32 or IMR32-CCT2 cells were collected in 100 μL of 5% FBS/PBS and spiked directly into CellSearch Autoprep tubes with 7.5 mL of blood from healthy human donors. Samples were then prepared and processed according to the CellSearch protocol. The antibody for CCT2-phycoerythrin (PE): [NP_006422] (LSBio) was added at a concentration of 24 μg/mL. Cells were analyzed through the CellSearch Analyzer II for CTCs and CCT2-positive (CCT2+) cancer cells as described previously (32). Blood collection was conducted by observing the human subject protection criteria for the University of Central Florida (UCF) and was approved by the Institutional Review Board (IRB) committees at UCF. Informed consent agreements were obtained from all participating subjects, at which time two 10 mLs of blood in CellSave tubes (Menarini) were drawn and the Federal Privacy Regulations for protected health information were followed by study personnel.

### Statistical analysis

Statistical analysis was carried out using GraphPad Prism 9 Software (GraphPad Prism Software Inc., San Diego, CA, USA). Bioinformatic statistical analyses were carried out using UCSC Xena and after downloading data from the UCSC Xena Data Hub, using R (version 4.1.2). For RT-qPCR analysis, ΔCT = (CT of target gene – CT of the endogenous gene (GAPDH)). Samples were normalized by dividing the sample’s ΔCT by the control sample’s ΔCT, with MDA-MB-231 as the control. To calculate relative gene expression, we used the equation RQ=2^-ΔCT^, using the normalized ΔCT values. For analyzing the significance of morphological effects of CT20p treatment, a two-tailed T-test with Welch’s correction was used at a confidence level of 95%.

## Results

### Data mining reveals that pediatric cancers have high levels of CCT2

**Figure 1:**
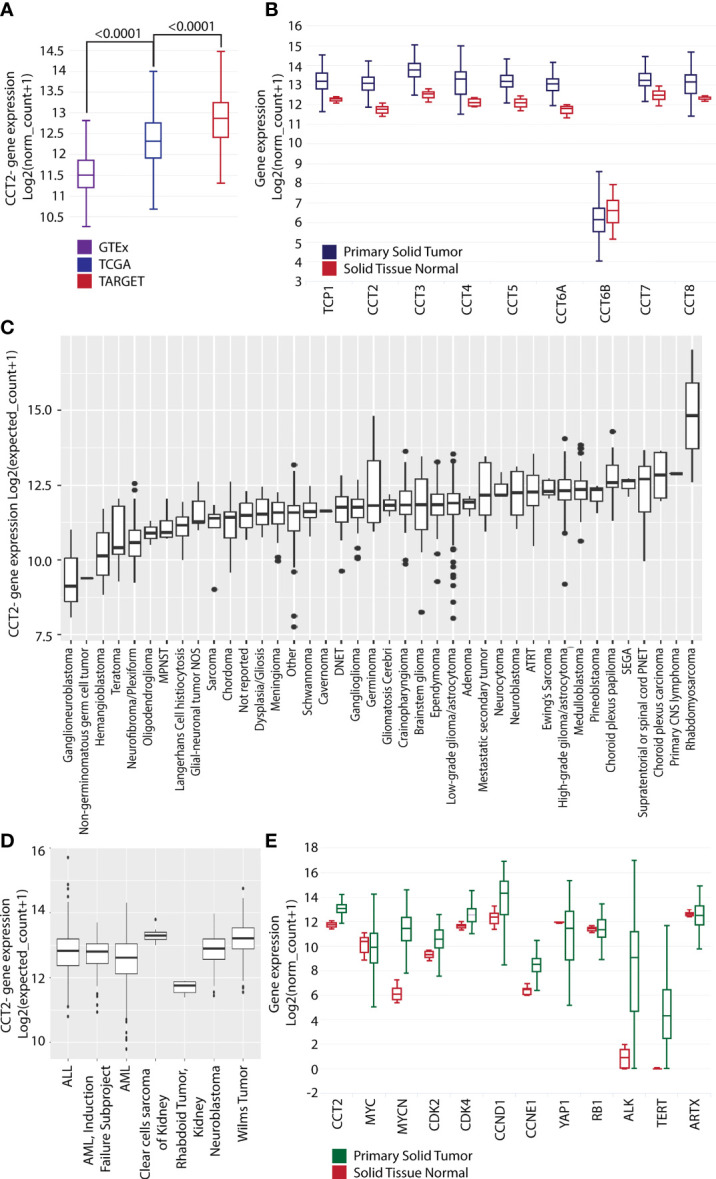
CCT2 expression is higher in primary solid tumors than in normal tissue. **(A)** UCSC Xena dataset GTEx (purple), TCGA (blue), and TARGET (red) cohort (n=19,131) comparing *CCT2* gene expression levels. **(B)** UCSC Xena TARGET Pan-Cancer dataset (n=297) primary solid tumor (blue) vs. solid tissue normal (red) looking at gene expression for all nine subunits of CCT. Statistical analysis with Welch’s t-test is as follows. TCP1: p < 0.0001 (t = 18.21); CCT2: p < 0.0001 (t = 19.27); CCT3: p < 0.0001 (t = 19.26); CCT4: p < 0.0001 (t = 16.69); CCT5: p < 0.0001 (t = 13.77); CCT6A: p < 0.0001 (t = 18.98); CCT6B: p = 0.1223 (t = -1.671); CCT7: p = < 0.0001 (t = 9.710); CCT8: p = < 0.0001 (t = 12.37). **(C)** KidsFirst/CBTTC cohort (n=961) comparing *CCT2* gene expression in specific pediatric cancers. ATRT: Atypical Teratoid Rhabdoid Tumor, DNET: Dysembryoplastic neuroepithelial tumor, MPNST: Malignant peripheral nerve sheath tumor, SEGA: Subependymal giant cell astrocytoma. Glioma/astrocytoma: High-grade (WHO grade III/IV) vs low-grade (WHO grade I/II). **(D)** UCSC Xena dataset TARGET (n=734) comparing *CCT2* gene expression in specific pediatric cancers. ALL: Acute Lymphoblastic Leukemia, AML: Acute Myeloid Leukemia. **(E)** UCSC Xena TARGET Pan-Cancer dataset (n=297) solid tissue normal (red) and primary solid tumor (green) looking at expression levels of nine genes: *CCT2*, *MYC*, *MYCN*, *CDK2*, *CDK4*, *CCND1*, *CCNE1*, *YAP1*, *RB1, ALK1, TERT, and ATRX*. Statistical analysis with Welch’s t-tests is as follows. CCT2: p < 0.0001 (t = 19.27); MYC: p = 0.3189 (t = -1.030); MYCN: p < 0.0001 (t = 24.27); CDK2: p < 0.0001 (t = 11.84); CDK4: p < 0.0001 (t = 12.72); CCND1: p < 0.0001 (t = 5.330); CCNE1: p < 0.0001 (t = 14.83); YAP1: p = < 0.0001 (t = -7.533); RB1: p = 0.8831 (t = -0.1478), ALK: p<0.0001 (t = -23.72), TERT: p<0.0001 (t = -26.45), ATRX p= 0.0918 (t = 1.714). The specific breakdown of the number of tumor tissues in each group is shown in **Table S3**. Boxplots presents five statistics – the minimum, the lower quartile (25%), the median, the upper quartile (75%), and the maximum. The box length indicates the sample variability (25% - 75%), and the line across the box shows the median. Dots present outliers.

Research from our lab and others showed that CCT2 is upregulated in multiple adult cancers (6). To investigate the relevance of CCT in pediatric cancers, we used the UCSC Xena database to compare cohorts, GTEx (normal tissues), TCGA (adult cancerous and normal tissues), and TARGET (pediatric cancerous and normal tissues) for gene expression of *CCT2*. The results indicated that TCGA samples had significantly higher expression levels of *CCT2* (<0.0001) than GTEx, and that pediatric TARGET samples had significantly higher expression levels of *CCT2* (<0.0001) than TCGA samples, **Figure 1A**. Expression of all eight CCT subunits was significantly higher in the primary solid tumor over normal solid tissue, except for *CCT6B*, which is found only in testes and is typically a negative marker for other tissues, **Figure 1B**. To identify which pediatric cancers might benefit from developing CCT2 as a molecular target, we analyzed the KidsFirst/CBTTC cohort (21 datasets) and the TARGET Pan-Cancer cohort (12 datasets) by cancer type. High expression of *CCT2* was noted in rhabdomyosarcoma, choroid plexus carcinoma, high-grade glioma/astrocytoma, medulloblastoma, metastatic secondary tumors, neuroblastomas, and others, **Figure 1C**. Interestingly, slow-growing or benign tumors, like hemangioblastoma, ganglioneuroblastomas, or neurofibroma plexiform had lower levels of *CCT2* expression, **Figure 1C**. Clear cell sarcoma of the kidney, Wilms tumor, and, again, neuroblastomas also had some of the highest levels of *CCT2* expression compared to other cancers, **Figure 1D**. The specific breakdown of the number of tumor tissues in each group is shown in **Table S3**. We then determined the relative expression of *CCT2* in comparison to other oncogenes, with a focus on neuroblastoma-relevant oncogenes, some of which are potential interactors with CCT (BioGRID). Expression levels of *CCT2, MYCN, CDK2, CDK4, CCND1, CCNE1*, and *ALK and TERT* showed significant increases in the primary solid tumor over normal solid tissue, while *MYC*, *YAP1*, *RB1, and ARTX* did not, **Figure 1E**. Importantly, among these markers, *MYCN* and *CCT2* expression showed the highest t-values, indicating a distinct difference between the two sample types, which suggests that the difference in *CCT2* gene expression between cancer and normal tissues is more likely to be true and reliable.

### CCT2 protein is detected in multiple pediatric cancers and neuroblastoma tissues

**Figure 2:**
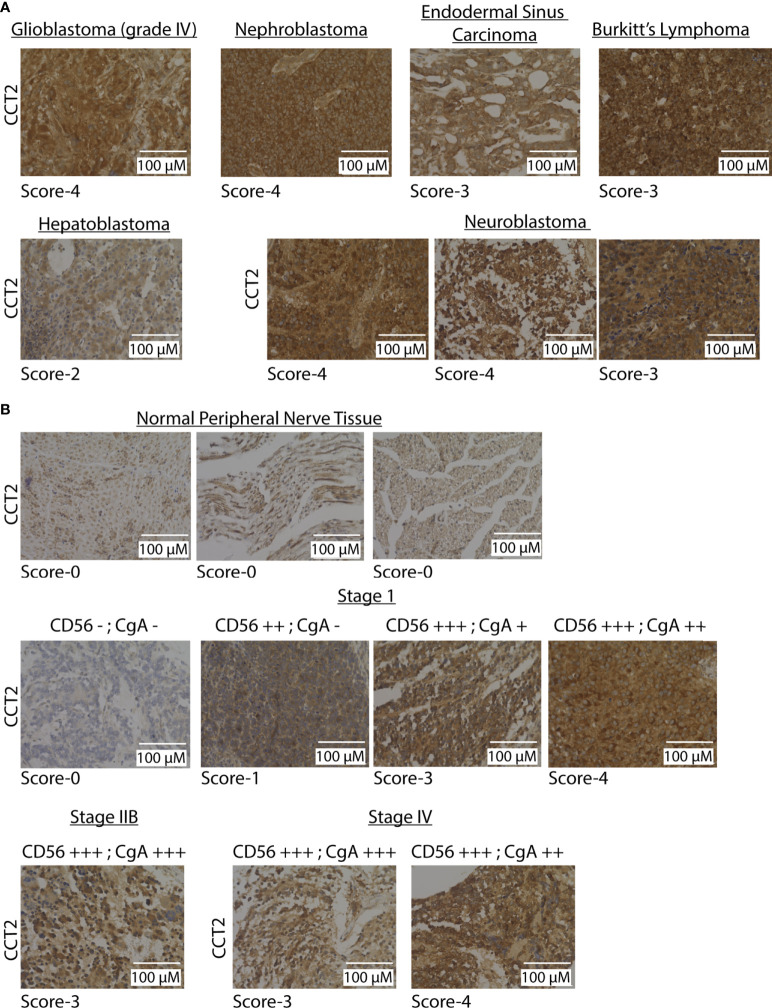
CCT2 is detected in pediatric tumor tissues. **(A)** Representative IHC images of CCT2 staining using the pediatric malignant tumor tissue array with normal tissue as control (PC701, US Biomax) (n=70). The CCT2 staining score is listed below each image. **(B)** Representative images from CCT2 staining using the neuroblastoma and peripheral nerve tissue microarray (NB642c, US Biomax) (n=32). CCT2 stain score is listed below each image as well as CD56 and CgA scores provided with TMA. All images were taken at 40x magnification.

To directly evaluate CCT2 levels in tumor tissues, we used a pediatric cancer tissue microarray (TMA) for examining CCT2 protein by standard IHC. A clinical/anatomic pathologist on our team previously developed an IHC scoring scheme from 0-4 for CCT2 staining of tissues, with normal tissues staining usually in the 0-1 range and tumor tissues staining >1, based on the intensity of the cytoplasmic and nuclear stains (21, 26, 32). Results from the TMA revealed strong CCT2 staining (scores 3-4) across most of the tumor tissues, representative images are shown in **Figure 2A**, with the full TMA shown in **Figure S2**. Most normal tissues had low staining (score 1-2) for CCT2 protein, **Figure S2**. Since neuroblastoma had some of the highest CCT2 protein levels, we evaluated CCT2 by IHC in a dedicated neuroblastoma TMA. CCT2 staining was present in all neuroblastoma tissues, with low staining evident in the normal peripheral nervous tissue, representative images are shown in **Figure 2B** with the full TMA shown in **Figure S3**. Over 60% of the neuroblastoma tumors had CCT2 staining scores of 3-4, **Table S4**. This data indicates that CCT2, while essential for most cells, is highly expressed in cancerous cells and that there is a detectable and measurable difference between normal and tumor tissues. The TMA also included information on tumor grade, cluster of differentiation 56 (CD56) staining, and chromogranin A (CgA), which are diagnostic markers for neuroblastoma. When we grouped the tumor tissues based on these markers, a trend was observed that increased CCT2 correlated with a higher incidence of one or more of these markers, which are indicative of cancer progression, **Figure 2B** and **Table S5**. While more tumor samples for each cancer stage are needed to achieve statistical significance, this data suggests that CCT2 levels may correlate with neuroblastoma progression and be a biological indicator of disseminated or invasive disease.

### CCT2 expression in neuroblastoma cells is comparable to metastatic cancer like TNBC

**Figure 3:**
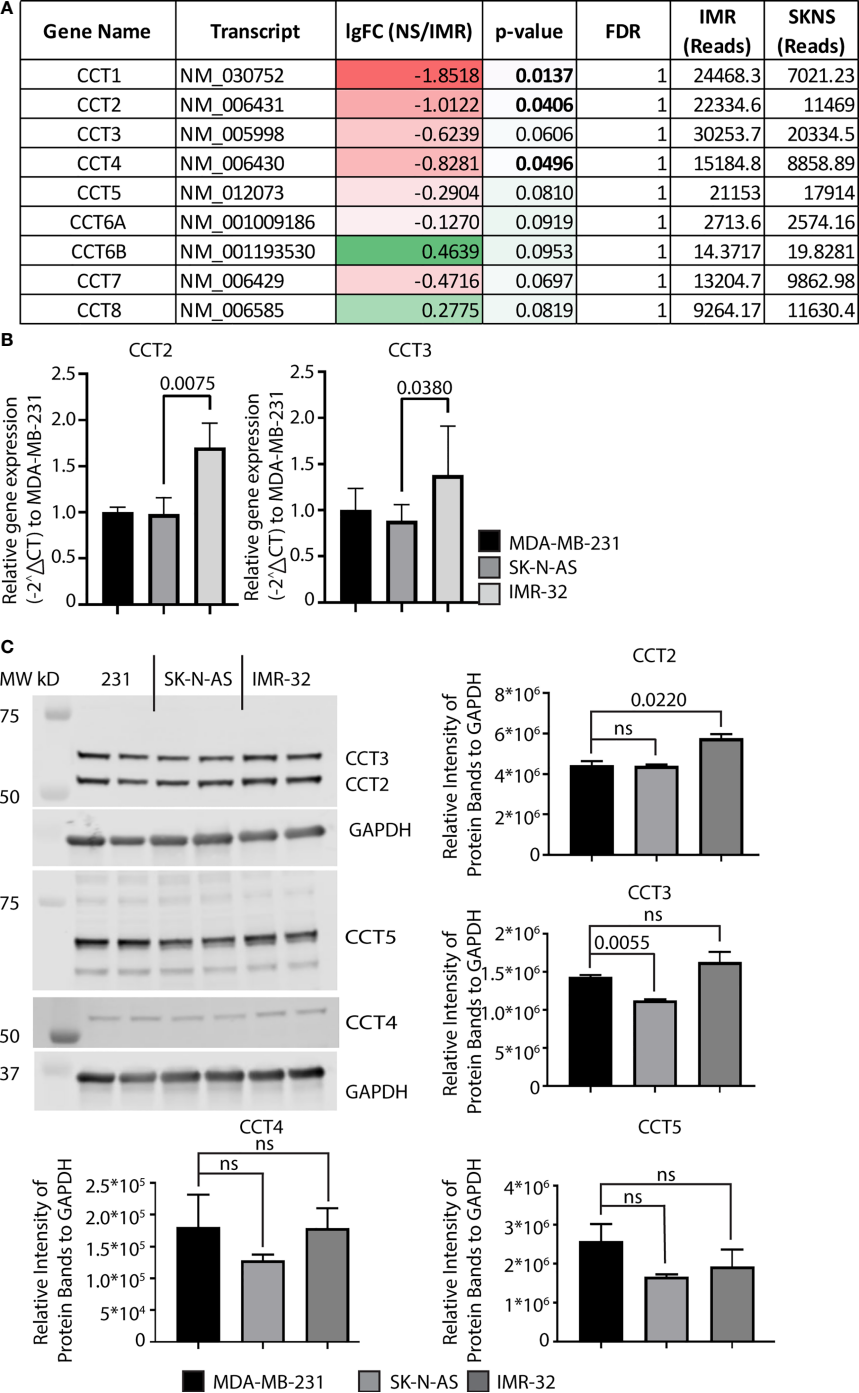
Expression of CCT subunits in neuroblastoma cell lines are comparable to metastatic TNBC cells. **(A)** RNA expression for the nine CCT subunits in IMR-32 and SK-N-AS cells was acquired from next-generation RNA sequencing data (33). **(B)** Relative mRNA expression for total CCT2 and CCT3 was determined by RT-qPCR (n=4). GAPDH was used as a reference gene. Calculations were based on using the 2^-ΔΔ CT^ equation. Results from MDA-MB-231 cells were used as a comparison. **(C)** Immunoblots for CCT2, CCT3, CCT4, and CCT5 were performed with specific antibodies as described in Methods using protein lysates prepared from SK-N-AS and IMR-32 cells. Representative blots are shown (n=2), data replicates were normalized to GAPDH, and the results are summarized in the graphs. p-values indicated in graphs; ns, not significant.

We next examined CCT2 expression using neuroblastoma cell lines. Starting with RNA-sequencing (RNA-seq) data from SK-N-AS and IMR-32 cell lines (36), we examined expression levels of all CCT subunits. IMR-32 and SK-N-AS cell lines were derived from high-risk neuroblastoma patients. However, IMR-32 cells are *MYCN*-amplified while SK-N-A cells are *MYCN* down-regulated (37). All CCT subunits, except for the negative control *CCT6B*, were high in both cell lines, while the expression of *CCT1, CCT2*, and *CCT4* was increased in IMR-32 compared to SK-N-AS cells, **Figure 3A**. Most notable was the robust number of reads for all CCT subunits across both cell lines compared to other genes. Typical reads for the average gene are around 300-1,000 reads; however, for CCT subunits, the reads ranged from 10,000–30,000, indicating that the chaperonin is highly expressed in both cell lines. We then extracted RNA and generated protein lysates from IMR-32 and SK-N-AS cell lines to measure baseline levels of RNA and protein for CCT subunits. For comparison, we used the highly metastatic TNBC cell line, MDA-MB-231, which our lab previously reported had high levels of CCT among breast cancer cell lines (7, 21). Results with the IMR-32 and SK-N-AS cells, showed that relative RNA levels of CCT2 and CCT3 (as a control for other CCT subunits) were comparable to MDA-MB-231 cells, if not higher (for IMR-32 cells), **Figure 3B**. Moreover, while the expression of CCT2 and CCT3 was high in both cell lines, it was significantly higher in IMR-32 cells compared to SK-N-AS cells (p=0.0075 and p=0.0380 respectively). Immunoblot analysis supported the RNA data, showing that protein levels for CCT2, CCT3, CCT4, and CCT5 were comparable in SK-N-AS and IMR-32 cells to the TNBC cells, **Figure 3C**. This data shows that CCT is highly expressed in both IMR-32 and SK-N-AS cell lines at levels similar to those found in metastatic TNBC cells, suggesting that the protein-folding activity of the chaperonin may enable the invasive behavior that enables the development of high-risk neuroblastomas.

### Exogenous expression of CCT2-FLAG negatively impacts the endogenous production of CCT2 in neuroblastoma cells

**Figure 4:**
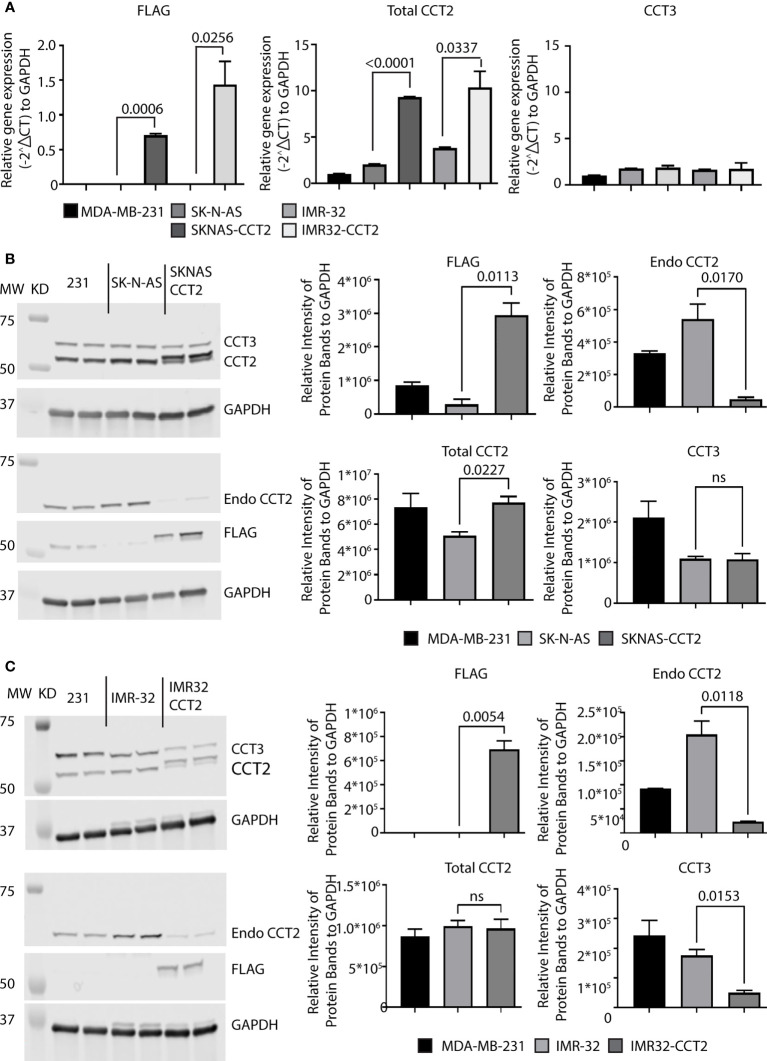
Exogenous expression of CCT2-FLAG in SK-N-AS and IMR-32 cells decreased endogenous CCT2. SK-N-AS and IMR-32 cells were transduced with lentiviral CCT2-FLAG and un-transduced cells were used as controls as described in Methods. **(A)** Relative mRNA expression for FLAG, total CCT2, and endogenous CCT3 in SK-N-AS, IMR-32, SKNAS-CCT2 and IMR32-CCT2 cells was determined by RT-qPCR (n=2). GAPDH was used as a reference gene. Calculations were based on using the 2^-ΔΔ CT^ equation. Results from MDA-MB-231 cells were used as a comparison. **(B, C)** Immunoblots for exogenous CCT2-FLAG (anti-FLAG antibody), total CCT2 (N-terminal specific anti-CCT2 antibody), endogenous CCT2 (C-terminal specific anti-CCT2 antibody), and endogenous CCT3 proteins using protein lysates from SK-N-AS and SKNAS-CCT2 cells **(B)** and IMR-32 and IMR32-CCT2 cells are shown **(C)**. Results from MDA-MB-231 cells are included for comparison. Representative blots are shown (n=2). Lanes: 1. and 2. MDA-MB-231, 3. and 4. SK-N-AS, 5. and 6. SKNAS-CCT2. **(C)** IMR-32 western blots for FLAG, Endo CCT2, Total CCT2 and CCT3 after lentiviral transduction. Lanes: 1. and 2. MDA-MB-231, 3. and 4. IMR-32, 5. and 6. IMR32-CCT2. Data replicates are summarized in the graphs and normalized to GAPDH. p-values indicated in graphs; ns, not significant.

Exogenously expressing CCT2 in early stage, luminal A breast cancer cells increased their proliferation and anchorage-independent growth (7, 8). However, unlike neuroblastoma cells, these breast cancer cells had low levels of CCT that were closer to normal breast epithelial cells than metastatic cells (21); hence increasing CCT2 changed their phenotypic behavior. We, therefore, determined if increasing exogenous CCT2 expression in SK-N-AS and IMR-32 cells would likewise alter the behavior of these cells, given their higher baseline levels of CCT2. SK-N-AS and IMR-32 cells were transfected with a lentiviral vector (LVV) to exogenously express a FLAG-tagged CCT2 construct (CCT2-FLAG) that we previously used in breast cancer cells (7). We opted for this approach given the efficiency of LVV gene transduction, with the tradeoff that insertional mutagenesis at the site of LVV chromosomal integration could disrupt the expression of normal genes, resulting in cells that are unusable as negative controls. Hence, as controls in our experiments, we used non-transduced cell lines. To minimize non-specific effects from the LVV transduction itself, we used SK-N-AS and IMR-32 cells that were transduced several months before experiments were performed and maintained as stable cell lines. RT-qPCR analysis showed that CCT2-FLAG mRNA was detected in both SKNAS-CCT2 and IMR32-CCT2 cell lines. Statistically significant increases were seen in total CCT2 RNA for SKNAS-CCT2 cells (p<0.0001) and IMR32-CCT2 cells (p=0.0337) compared to control cells, **Figure 4A**. Increases in CCT3 RNA were not significant for either cell line, **Figure 4A**. To determine if increased gene expression led to increased protein, we performed immunoblots for the FLAG tag, endogenous CCT2 (using a C-terminal specific antibody), and total CCT2 (using an N-terminal specific antibody), and total CCT3. Expression of CCT2-FLAG in SKNAS-CCT2 cells resulted in detection of the FLAG tag (p=0.0113) and increased total CCT2 by 52% (p=0.0227) over the control SK-N-AS cells, but it also caused a significant decrease in endogenous CCT2 by 91% (p=0.0170), **Figure 4B**. Hence most of the CCT2 available for forming the CCT complex in SKNAS-CCT2 cells was the CCT2-FLAG. We previously showed in breast cancer cells expressing CCT2-FLAG that ~70% of the CCT2-FLAG protein was incorporated into the CCT hetero-oligomeric complex and ~30% remained as an unincorporated monomer (7, 8). While total CCT2 protein levels increased in the SKNAS-CCT2 cells, total CCT3 protein levels remained comparable to control SK-N-AS cells, **Figure 4B**. Expression of CCT2-FLAG in IMR-32 cells showed detectable expression of the FLAG-tagged protein compared to control cells (p=0.0054), although a log-fold lower than in the SKNAS-CCT2 cells, **Figures 4B, C**. While this could be due to differences in transduction efficiency between the cell lines, there are also may be differences in the inherent regulatory mechanisms that control CCT subunit protein levels in these cells. As with the SKNAS-CCT2 cells, we noted a significant decrease in endogenous CCT2 by 88% (p=0.0118) in the IMR32-CCT2 cells, **Figure 4C**. CCT subunits can be degraded by the proteasome (29), hence the loss of endogenous CCT2 upon expression of exogenous CC2-FLAG could be due to the degradation of CCT2 monomers through a yet-to-be-determined feedback mechanism. Such a mechanism could also explain the decrease in endogenous CCT3 noted in IMR32-CCT2 cells. While total CCT2 levels did not increase in IMR32-CCT2 cells compared to control cells, these cells maintained a continuous supply of exogenous CCT2-FLAG that could function in place in the endogenous CCT2 protein and drive changes in cell behavior, **Figure 4C**.

### Expression of CCT2-FLAG increases actin and tubulin and enhances migration of neuroblastoma cells

**Figure 5:**
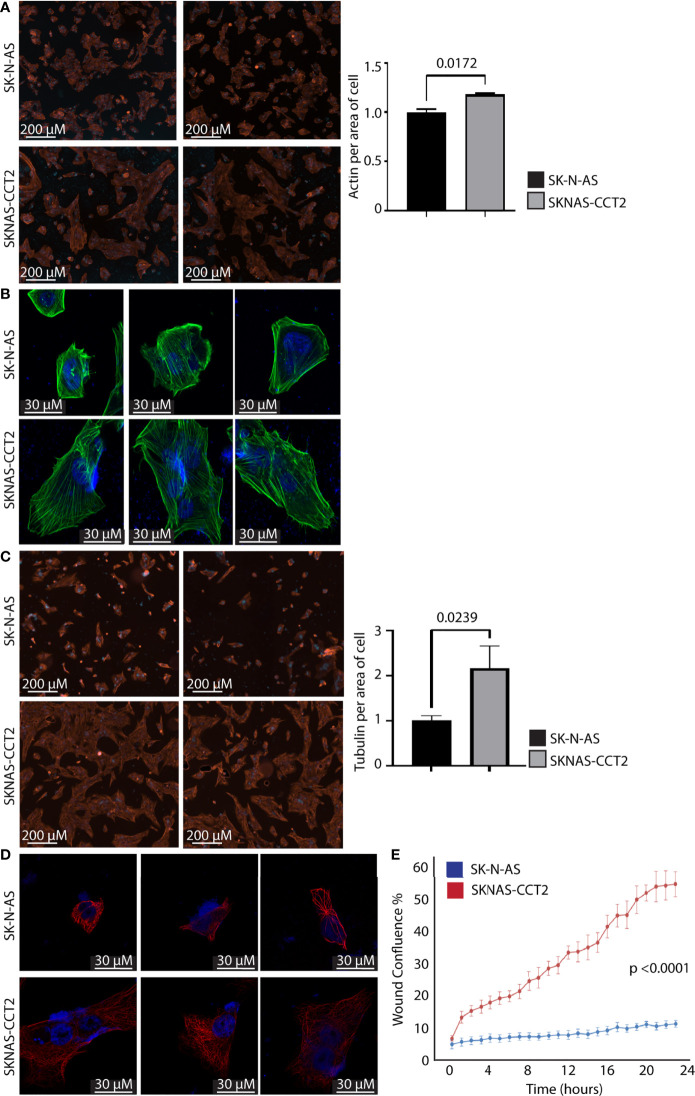
Exogenous expression of CCT2-FLAG increases actin and tubulin and enhances migration of SK-N-AS cells. **(A)** SKNAS-CCT2 and control SK-N-AS cells were stained for F-actin (ActinRed 555 ReadyProbes Reagent) and DAPI and representative images were acquired (ImageXpress Pico) at 20X magnification. Quantitation of fluorescent signal per average positive cell area is shown in the graph. **(B)** Cells from **(A)** were imaged by confocal microscopy at 100X magnification (Zeiss LSM 710). **(C)** SKNAS-CCT2 and control SK-N-AS cells were stained for tubulin using anti-α tubulin antibody (DM1A) and DAPI and representative images were acquired (ImageXpress Pico) at 20X magnification. Quantitation of fluorescent signal per average positive cell area is shown in the graph. **(D)** Cells from **(C)** were imaged by confocal microscopy at 100X magnification (Zeiss LSM 710). **(E)** A Scratch Wound Assay was performed to assess cell migration (Incucyte S3) over 24 hours using SK-N-AS and SKNAS-CCT2 cells (n=4). Percent closure or wound confluency is shown. p-values are indicated in graphs.

The protein-folding activity of CCT that supports cell migration is in part linked to generating the functional forms of its two obligate substrates: actin and tubulin from transcribed proteins. To investigate this, we imaged and quantified the polymerizing forms of actin and tubulin in SKNAS-CCT2 and IMR32-CCT2 cells compared to control cells. We observed significant morphological changes in SKNAS-CCT2 cells that correlated with increased total cell area containing F-actin, **Figures 5A, B**, and tubulin, **Figures 5C, D**. F-actin was visualized using phalloidin to stain actin filaments. Two representative fields of view illustrate F-actin staining by digital microscopy for SKNAS-CCT2 and control SK-N-AS cells. Quantitation of multiple fields revealed a statistically significant increase in the total cell area positive for F-actin in SKNAS-CCT2 cells compared to control cells, **Figure 5A**. SKNAS-CCT2 cells were larger and displayed partial polarization of F-actin, suggestive of increased movement, **Figure 5B**. The confocal images of actin filaments further highlighted these morphological changes, showing a wider dispersion of the actin filaments in a complex array in cells with CCT2-FLAG expression, as well as increased multinucleation in some cells. Tubulin was stained using an anti-α tubulin primary antibody (DM1A) and our results showed similar morphologic changes to those seen with F-actin, **Figure 5C**. Quantification of the SKNAS-CCT2 cells also revealed a statistically significant increase in the total cell area positive for tubulin compared to control cells, **Figure 5C**. A more complex network of microtubules was evident in the confocal images from SKNAS-CCT2 cells compared to SK-N-AS cells, **Figure 5D**. A possible functional outcome of increased F-actin and tubulin per average cell area could be increased migration. To measure this, we conducted a live cell scratch/wound assay, using the Incucyte system to monitor cell migration, comparing CCT2-FLAG expressing cells to control cells. Over 24 hours the rate of wound closure was visually assessed every hour. Compiled results showed a 5-fold increase (p<0.0001) in migration of SKNAS-CCT2 cells compared to controls, even at early timepoints before cellular replication, **Figure 5E**.

**Figure 6:**
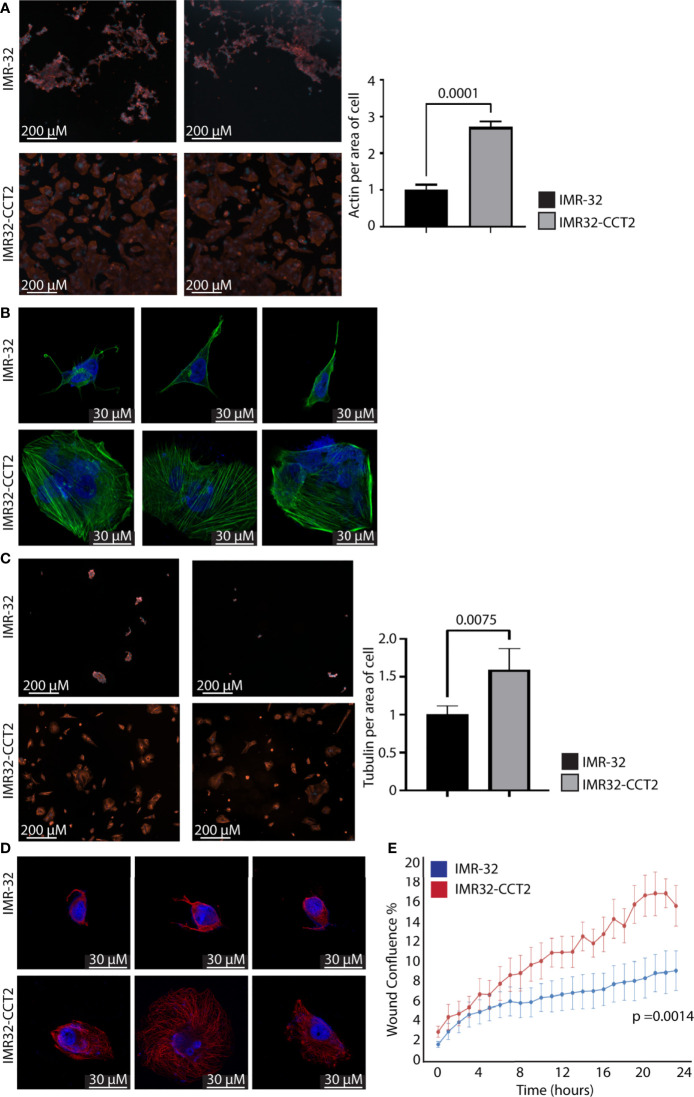
Exogenous expression of CCT2-FLAG increases actin and tubulin and enhances migration of IMR-32 cells. **(A)** IMR32-CCT2 and control IMR-32 cells were stained for F-actin (ActinRed 555 ReadyProbes Reagent) and DAPI and representative images were acquired (ImageXpress Pico) at 20X magnification. Quantitation of fluorescent signal per average positive cell area is shown in the graph. **(B)** Cells from **(A)** were imaged by confocal microscopy at 100X magnification (Zeiss LSM 710). **(C)** IMR32-CCT2 and control IMR-32 cells were stained for tubulin using anti-α tubulin antibody (DM1A) and DAPI and representative images were acquired (ImageXpress Pico) at 20X magnification. Quantitation of fluorescent signal per average positive cell area is shown in the graph. **(D)** Cells from **(C)** were imaged by confocal microscopy at 100X magnification (Zeiss LSM 710). **(E)** A Scratch Wound Assay was performed to assess cell migration (Incucyte S3) over 24 hours using IMR-32 and IMR32-CCT2 cells (n=4). Percent closure or wound confluency is shown. p-values are indicated in graphs.

A similar set of experiments were performed with IMR32-CCT2 cells comparing our findings to control IMR-32 cells. IMR32-CCT2 cells underwent significant morphological changes, such as increased cell spreading, in part due to more F-actin per cell area, **Figure 6A**. Confocal images of IMR32-CCT2 cells revealed larger cells with partially polarized arrays of actin microfilaments as compared to control IMR-32 cells, **Figure 6B**. Likewise, tubulin staining highlighted the increased cell area and morphologic changes in IMR32-CCT2 cells compared to controls, **Figure 6C**. A notable observation was that IMR-32 control cells tended to display low plating efficiency and were easily washed off due to poor adherence to culture plate surfaces, while the IMR32-CCT2 cells were more robust and adherent and more amenable to effective plating, likely a consequence of the cytoskeletal changes noted above. Confocal images revealed a more complex microtubule network in IMR32-CCT2 cells compared to control IMR32-cells, **Figures 6D**. While IMR-32 control cells are inherently a more migratory cell line compared to SK-N-AS cells (see baseline migration of SK-N-AS and IMR-32 cells, **Figures 5E**, **6E**), expressing CCT2-FLAG further increased their migratory capacity as shown in a scratch/wound assay visualized with the Incucyte system. A 2-fold increase (p=0.0014) in migration between IMR32-CCT2 and control cells was evident, **Figure 6E**. These results indicate that even in cells that are highly invasive like IMR-32, increasing CCT2 can enhance their migratory behavior which could be a contributing factor to producing the aggressive cancer cells that cause disseminated disease.

### Depletion of CCT2 in neuroblastoma cells decreases viability and migration

**Figure 7:**
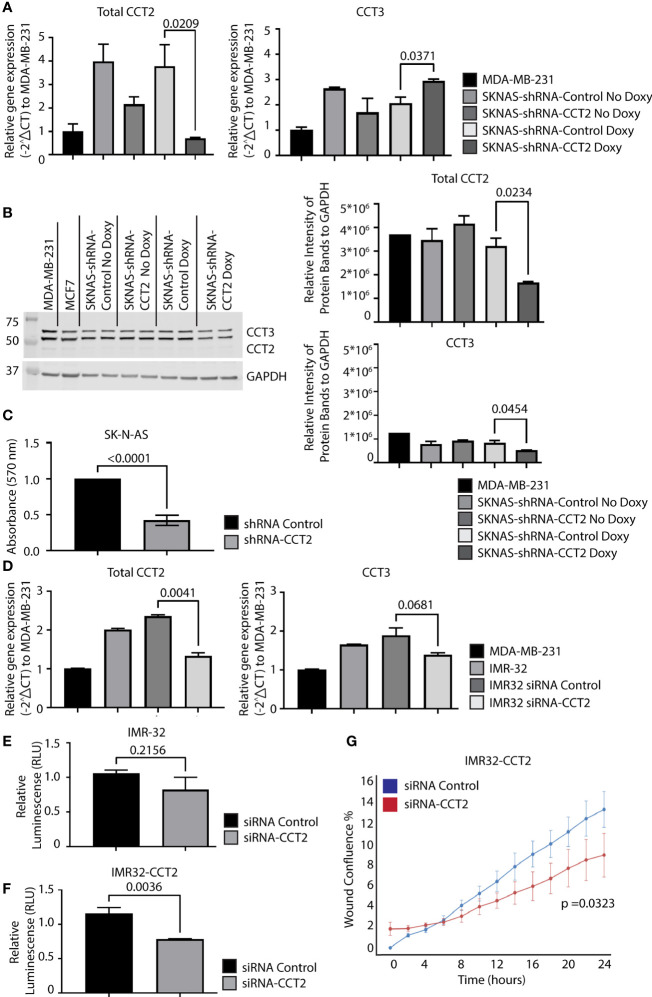
Depletion of CCT2 in SK-N-AS and IMR-32 cells reduces cell viability and reverses the effects of exogenous expression of CCT2-FLAG. **(A)** RNA was isolated from SK-N-AS cells stably expressing inducible CCT2-shRNA or control-shRNA lentiviral vectors with or without induction using 1 mg/mL doxycycline (Doxy) after 48 hours. Relative mRNA expression for total CCT2 and endogenous CCT3 was determined by RT-qPCR (n=2). GAPDH was used as a reference gene. Calculations were based on using the 2^-ΔΔ CT^ equation. Results from MDA-MB-231 cells were used for comparison. **(B)** Immunoblots for CCT2 and CCT3 were performed using protein lysates prepared from SK-N-AS cells stably expressing CCT2-shRNA or control-shRNA and induced with 1 mg/mL Doxy after 96 hours. A representative blot is shown (n=2), and data replicates were normalized to GAPDH, and the results are summarized in the graphs. **(C)** SK-N-AS cells were transiently transduced with lentiviral CCT2-shRNA or control shRNA and viability was determined after 96 hours using an MTT viability assay. **(D)** IMR-32 cells were treated with ON-TARGETplus Human CCT2 siRNA (Dharmacon), RNA extracted, and RT-qPCR performed for CCT2 and CCT3 as above. GAPDH was used as a reference gene. Results were normalized to MDA-MB-231 cells. **(E, F)** Viability of IMR-32 cells treated with either siRNA-CCT2 or control siRNA was assessed by CellTiter-Glo 2.0 Assay after 96 hours of treatment. **(G)** IMR32-CCT2 cells were treated with either siRNA-CCT2 or control siRNA and migration was assessed using the Incucyte scratch/wound assay. Wound closure was assessed at hourly intervals for 48 hours, with 0 hours starting 48 hours after siRNA treatment. p-values are indicated in graphs.

Inhibition of CCT2, using doxycycline (doxy) inducible system to express CCT2 small hairpin RNAs (shRNA), in a syngeneic TNBC mouse model prevented tumor growth (7) and impaired spheroid formation (8). To determine whether CCT2 depletion had a similar effect on neuroblastoma cells, we used the same CCT2 shRNA system that is induced by the addition of doxy. We first examined the efficiency of CCT2 depletion in SK-N-AS cells by stably transducing the cells with the lentiviral inducible vector for CCT2-shRNA or control shRNA. We found that 1 μg/mL of doxy induced CCT2-shRNA and caused a ~70% decrease in CCT2 RNA in SK-N-AS cells, **Figure 7A**. Under these conditions, CCT3 RNA levels slightly increased, perhaps as a compensatory mechanism, **Figure 7A**. Next, we examined CCT2 and CCT3 protein levels in the CCT2 depleted SK-N-AS cells and noted that a 48% loss of CCT2 protein was achieved along with a 36% decrease of CCT3, **Figure 7B**. To determine whether this amount of CCT2 depletion could affect cell viability, we used the same CCT2 and control-shRNAs, expressed in a non-inducible lentiviral vector, in a transient transduction experiment and assessed viability using a standard MTT assay. After 96 hrs of CCT2 or control shRNA treatment, we observed a 58% decrease in viability due to the CCT2 depletion as compared to control shRNA treatment, **Figure 7C**. It is worth noting that we had achieved comparable results with the same CCT2-shRNA in breast cancer cells, in which 50% depletion of CCT2 resulted in ~40% loss of cell viability (7).

Unlike the SK-N-AS cells, the IMR-32 cells were resistant to transduction with the lentiviral shRNA vectors and selection, likely due to their poor plating efficiency and low adherence. To determine whether IMR-32 cells were susceptible to CCT2 depletion, we used a different approach from the SK-N-AS cells, involving the transient transfection with a pool of four CCT2 targeting siRNAs. Using the CCT2 siRNAs, we achieved a ~40% decrease in CCT2 RNA and a slight but not statistically significant decrease in CCT3, **Figure 7D**. Because of low adherence, we could not perform the standard MTT assay with IMR-32 cells and instead used a luminescence-based ATP assay to assess viability. We found that ~40% depletion of CCT2 RNA in IMR-32 cells caused 22% cell death which was not statistically significant, **Figure 7E**. However, we repeated this experiment using the IMR32-CCT2 cells. Recall that these cells are larger, plate more efficiently, and adhere to surfaces better than the original cell line. Treatment with the CCT2 siRNAs in IMR32-CCT2 cells did induce ~33% cell death, likely a result of CCT2 depletion, **Figure 7F**. Importantly, when we tested the effect of this partial level of CCT2 depletion in IMR32-CCT2 cells, we noted a significant decrease (p=0.0323) in cell migration using the Incucyte scratch/wound assay and software, reversing the pro-migratory effects of exogenous CCT2-FLAG expression, **Figure 7G**. These results validate CCT2 as a therapeutic target for neuroblastoma, demonstrating that even partial depletion of CCT2 had a significant impact on the viability and cell migration under cell culture conditions.

### CT20p is a CCT inhibitor that targets the chaperonin complex and is cytotoxic to neuroblastoma cells

**Figure 8:**
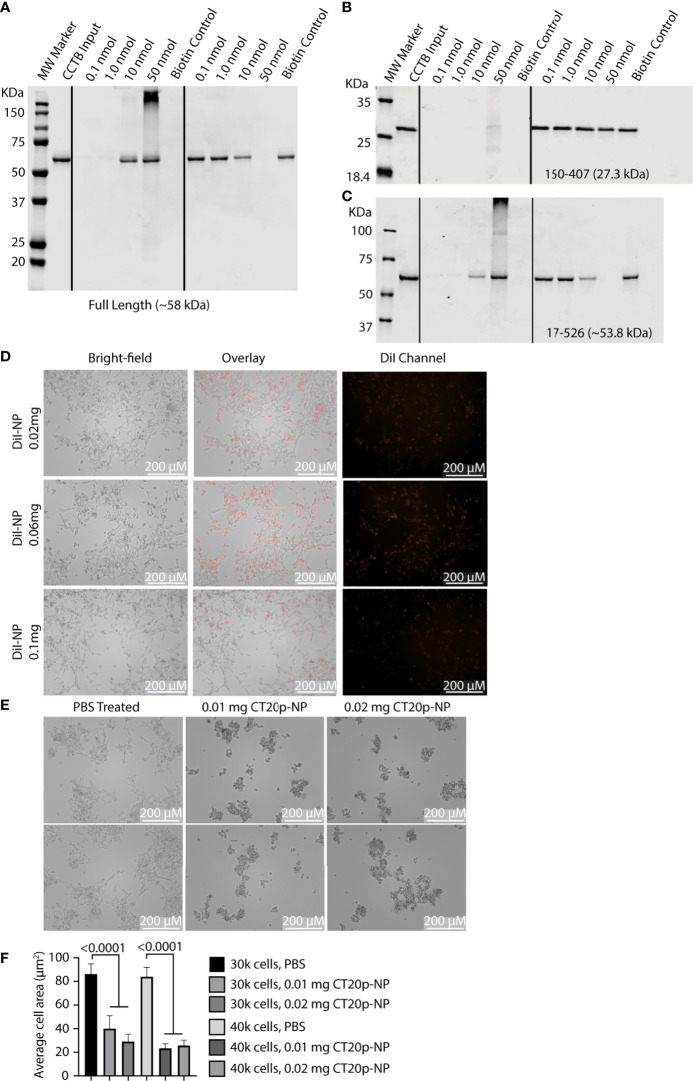
CT20p directly binds CCT and kills IMR-32 cells. **(A–C)** Full-length and truncated CCT2 recombinant proteins (0.1 nmol) were incubated with increasing amounts of biotinylated CT20p (0.1-50 nmol). Results from the “pull-down” were analyzed by immunoblotting for CCT2 as previously described (21). **(D)** IMR-32 cells were treated for 24 hours with PBS or NP loaded with the dye, DiI (DiI-NP) at 0.02-0.1 mg doses. Red fluorescence within cells indicates uptake of NP. Fluorescence and brightfield mages were acquired with Cytation 5 Cell Imaging Multi-Mode Reader at 10X magnification. **(E)** IMR-32 were treated with PBS or CT20p-NP at 0.01 mg or 0.02 mg doses and imaged as indicated above. **(F)** Quantification of cell death due to treatment with CT20p-NP was assessed by determining individual cell areas using Gen5 software.

Depletion of CCT2 showed that targeting one chaperonin subunit could affect the activity of the entire complex. However, there could also be unintended or compensatory effects on other subunits, such as what we observed for non-targeted subunits like CCT3 in IMR32-CCT2 cells. A preferred way to therapeutically target CCT would be using an inhibitor that impedes the chaperonin’s activity without directly affecting CCT subunit synthesis. Our lab previously discovered a 20 amino acid amphipathic peptide (having hydrophobic and hydrophilic parts) derived from the C-terminus of the apoptotic protein, Bax, that has inherent cytotoxic activity in cancer cells (34, 38). We called the peptide CT20p and developed a polymeric nanoparticle (NP) delivery system to transport the peptide through the vasculature into tumors (39, 40). The mechanism of action for CT20p was determined to be inhibition of CCT by performing a pull-down experiment with biotinylated CT20p and using a proteomics-based approach to identify the intracellular proteins that interacted with the biotinylated peptide. Seven of eight CCT subunits associated directly with CT20p and CCT2 were confirmed to bind CT20p in subsequent biochemical experiments using biotinylated CT20p and recombinant full-length CCT2 protein (21), **Figure 8A**. Since the association of CCT with substrates involves patterns of hydrophobic or polar residues (41, 42), the amphipathic nature of CT20p mimics these patterns, enabling the binding to CCT that depends not on individual amino acids but rather on the arrangement of hydrophobic and polar residues that is similar to the interaction of CCT with substrates. In support, we demonstrated that the cytotoxic action of CT20p was dependent on CCT by showing that cells with low levels of CCT or that were depleted for CCT2 exhibited resistance to CT20p, and cells in which CCT was highly expressed were sensitive to CT20p (21). To determine where on CCT subunits CT20p bound, we performed a simulation modeling experiment using Molecular Operating Environment (MOE) platform to determine the most energetically feasible binding site for CT20p on CCT2, as a representative CCT subunit, **Figure S1B**. We found that CT20p bound to a region in the equatorial domain of CCT2, which contains the ATP binding site and sites for inter- and intra-ring interactions. We confirmed this in a pull-down experiment, using a truncated form of recombinant CCT2 protein that was missing the first N-terminal 150 amino acids and last 120 C-terminal amino acids (CCT2-150-407) that form the equatorial domain of the subunit. Biotinylated CT20p did not bind to CCT2 150-407, while a second truncated version of recombinant CCT2 protein that retained more of the equatorial domain, (CCT2 17-526) did bind to biotinylated CT20p, **Figures 8B, C**. These results suggest that CT20p likely binds to a region on CCT2 (and possibly other subunits) that contains most of the equatorial domain of the subunit, potentially blocking sites for intra- and inter-ring contacts between subunits as well as portions of the nucleotide or ATP binding site, including catalytic aspartate residues (6).

To test whether CT20p proved cytotoxic with IMR-32 cells, we first determined that these cells could readily take up the carboxylated form of the polymeric NP carrier without the need for surface modifications like the addition of cancer-targeting ligands. Using NP loaded with the lipophilic dye, DiI, we tested three doses of DiI-NP (0.02, 0.06, and 0.1 mg) and showed that uptake of NP was achievable in IMR-32 cells with all doses tested and that minimal non-specific cell death was observed only at the highest dose, **Figure 8D**. We then treated the IMR-32 cells with CT20p-NP at two doses (0.01 and 0.02 mg) and observed cell death within 24-hrs as shown by representative images of aggregated clumps of dead/dying cells, **Figure 8E**, and reduced cell numbers that were indicative of cells detaching and dying, **Figure 8F**. It should be noted that IMR-32 cells treated with CT20p rounded up, detached, and died upon uptake of CT20p-NP in a manner consistent with our previous observations of CT20p-NP in the treatment of cultured cancer cells (34) and matches what we observed in breast and prostate cancer xenograft models (21, 26).

### IMR-32 cells are detectable in healthy human blood using CCT2 for identification

**Figure 9:**
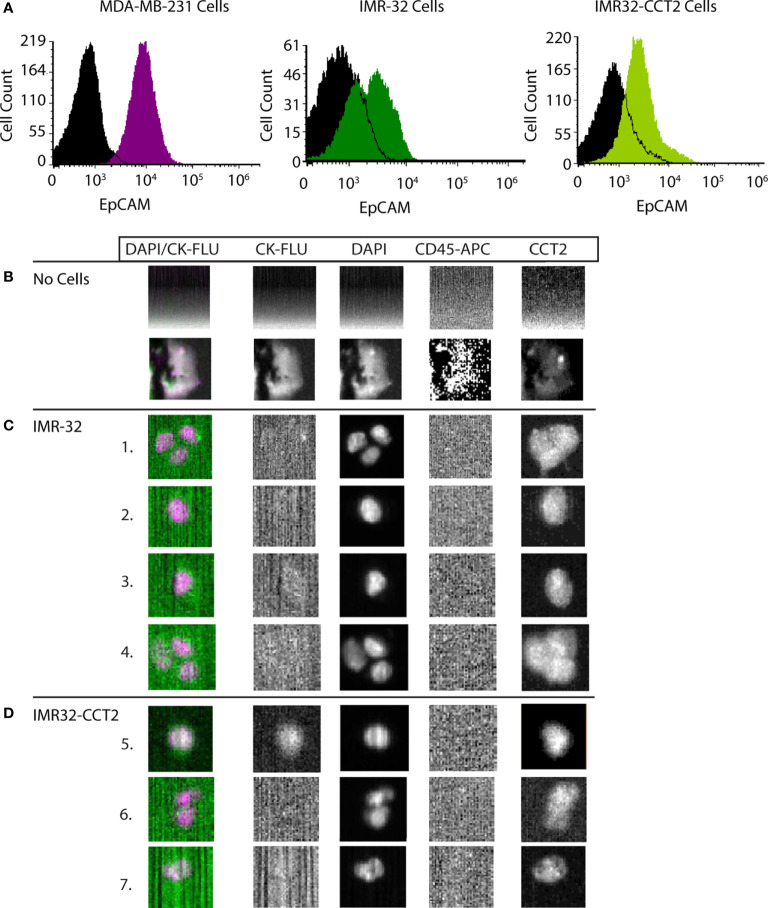
CCT2 enables the detection of neuroblastoma cells spiked into blood. **(A)** Levels of EpCAM on cell membranes of IMR-32 and IMR32-CCT2 cells were determined by flow cytometry using a specific antibody for EPCAM. IMR-32 cells: EpCAM (green) and isotype control (black). IMR32-CCT2 cells: EpCAM (light green) and isotype control (black). EpCAM levels (purple) compared to isotype control (black) on MDA-MB-231 cells are shown for comparison. **(B)** Representative images of human blood without spiked cancer cells. **(C)** IMR-32 cells were spiked into human blood. Representative images of IMR-32 cells recovered using the CCT2-optimized CellSearch protocol are shown. **(D)** IMR32-CCT2 cells were spiked into human blood. Representative images of IMR32-CCT2 cells recovered using the CCT2-optimized CellSearch protocol are shown. The first column is an overlay of DAPI (column three) and cytokeratin or CK-FLU (column two), column four is the CD45 marker for leukocytes and column five is the signal for CCT2, detected using an anti-CCT2-PE antibody.

Given that our data support CCT as an essential enabler of the migratory phenotype in SK-N-AS and IMR-32 cells, we tested whether CCT2 could be used as a diagnostic indicator for invasive neuroblastoma cells. We opted to use a liquid biopsy approach by detecting and enumerating CTCs using the CellSearch System. CellSearch is an FDA-approved enrichment method for enumeration of CTCs in the prognosis and monitoring of breast, colon, and prostate cancer (43, 44). Previously our lab modified the CellSearch CXC kit to incorporate the intracellular staining of CCT2 in the automated protocol that stains for cytokeratins 8, 18, and 19 (CKs), CD45, and DAPI (32). The CellSearch System uses ferrofluid or iron particles conjugated to an anti-epithelial cellular adhesion molecule (EpCAM) antibody to magnetically capture and initially enrich CTCs from blood. This is followed by staining and identification of CTCs using CKs and DAPI. The CellSearch System criteria screens for cells that are positive for CKs (a marker for epithelial carcinomas), negative for CD45 (not a blood leukocyte), and DAPI positive (a cell with an intact nucleus). To use the CellSearch CXC kit for investigating CCT2 in the detection of neuroblastoma cells shed in blood, we first confirmed that the IMR-32 cells express EpCAM. We found that IMR-32 and IMR-32-CCT2 cells have detectable, albeit low, EpCAM levels, **Figure 9A**. We previously noted that MDA-MB-231 cells also had low EpCAM levels but could be used in CellSearch System to detect cells spiked into blood (32). EpCAM-low cells can be processed with CellSearch System because the anti-EpCAM ferrofluid used in the first step of the protocol enables controlled aggregation so that cells with even low EpCAM levels are captured and enriched for downstream processing. We next determined that IMR-32 cells could be intracellularly stained for CCT2 and visualized by the CellSearch System (Analyzer II) by using a manual staining technique in place of the automated staining in the AutoPrep (32, 35). We assessed different concentrations of anti-CCT2 antibodies to determine the optimal conditions for staining of IMR-32 cells. Results from manual stain revealed that most of the IMR-32 cells were not positive for CKs, which is consistent with the fact that IMR-32 cells are not of epithelial origin, **Figure S4**. Manual staining confirmed that 24 μg/mL of CCT2 antibody was optimal, therefore we used this antibody concentration moving forward.

We spiked 7.5 mLs of blood from healthy human donors with ~200,000 IMR-32 or IMR32-CCT2 cells and stained for CCT2 using our modified CellSearch CXC kit protocol. Note that in the absence of spiked IMR-32 cells, background debris is mainly detected in the ‘blood only’ sample, representative images shown in **Figure 9B** (32). In the ‘blood with spiked cells’, few IMR-32 cells were detected by the CellSearch Analyzer using the standard CellSearch criteria for CTCs (above) due to the absence of positive CK staining (only one cell was observed in the IMR32-CCT2 cells), **Figures 9C, D**. However, when using CCT2 in place of CKs to detect cells, we were able to visualize the IMR-32 cells spiked into blood cells, **Figures 9C, D**. These cells met the remaining criteria of the CellSearch protocol for CTCs, including staining CD45 negative (hence not blood leukocytes) and DAPI positive (confirming they are cells with an intact nucleus). Our results demonstrate that CCT2 could be used as a diagnostic indicator for malignant neuroblastoma cells shed into blood, using liquid biopsy protocols like the enumeration of CTCs with the CellSearch System.

## Discussion

Neuroblastoma is a childhood cancer in which approximately 50% of patients are classified with high-risk disease. Using two neuroblastoma cell lines, SK-N-AS and IMR-32, derived from patients with bone and abdominal metastases, respectively, we investigated the role of the CCT chaperonin as an enabler of a migratory phenotype in these cells. A bioinformatics approach using adult and pediatric cancer databases showed that expression of *CCT* subunits was increased in pediatric cancers over normal tissues, which was confirmed with histologic data using pediatric cancer TMAs. Among the pediatric cancers, neuroblastomas emerged as a cancer type with high expression levels of CCT subunits. Both SK-N-AS and IMR-32 cultured cells had baseline levels of CCT subunits that were comparable if not higher than a metastatic TNBC cell line. To establish that CCT was essential for the migration of SK-N-AS and IMR-32 cells, we expressed a CCT2-FLAG construct and observed increases in CCT2 RNA. But exogenously expressing CCT2 also deregulated the expression of the endogenous CCT2 protein, alluding to the presence of an unknown compensatory mechanism that may control the size of the CCT subunit monomer pool. Despite this, expression of CCT2-FLAG significantly altered cell morphology, with both SKNAS-CCT2 cells and IMR32-CCT2 cells displaying expansive cytoskeletal alterations due to the increased abundance of the cytoskeletal building blocks, actin and tubulin, per average cell area. Since actin and tubulin are obligate clients of the CCT protein folding complex, it is significant that exogenously expressing one of the eight subunits that form the CCT heterooligomer was sufficient to increase the chaperonin’s protein folding activity. A probable result of supplying cells with more functional actin and tubulin was increased migration of SKNAS-CCT2 and IMR32-CCT2 cells that were dependent on CCT2. Depletion of CCT2 reversed these effects and reduced cell viability. To advance the use of CCT as a molecular target for neuroblastoma, we tested a CCT inhibitor developed by the Khaled lab, CT20p, and demonstrated its efficacy in killing neuroblastoma cells. We further showed that CCT2 could be used as a diagnostic marker to identify neuroblastoma cells shed into blood. This is important because current markers for CTCs are based on the detection of epithelial-based markers like CKs that may not be present in neuroblastoma cells, especially those with mesenchymal cell features.

Neuroblastomas are characterized by intratumoral heterogeneity that remains poorly understood. Such tumors are mainly characterized by genomic amplification events that produce multiple copies of *MYCN* that in total increase MYCN expression. Few somatic mutations are associated with neuroblastoma other than *ALK* (anaplastic lymphoma kinase) found in 8-10% of neuroblastoma cases. In about 4% of spontaneous neuroblastoma cases, loss-of-function mutations in *PHOX2B* (paired-like homeobox 2B) can occur. In the high-risk neuroblastoma genome, there are few additional recurrent mutations other than *ATRX* (7.1% deletion, 2.5% mutation), or *PTPN11* (2.9%) (45). To address this therapeutic challenge caused by clinical heterogeneity that is not explained at the genomic level, studies of super-enhancer-associated transcription factor networks revealed different subtypes of neuroblastoma cell lines, termed mesenchymal (MES) or adrenergic (ADRN). Neuroblastoma cells could be distinguished by belonging to either group. For example, the SK-N-AS cells are considered MES cells, while the IMR-32 are ADRN cells. This is similar to the idea of the epithelial to mesenchymal transition (EMT) states. However, while neuroblastoma cells can be classified as either in the MES or ADRN state, sufficient plasticity exists where transitioning between states could also cause transcriptional changes that are independent of the cell’s mutational status (46). Expanded complexity of transcriptional networks is exemplified with MES subtypes that can include both *MYCN*-amplified and nonamplified, high-risk or low-risk, and may also overlap with the ADRN group (47). Further, changes in transcriptional networks that alter cell phenotypes can occur during the development of drug resistance, both as a consequence of targeted therapies that alter gene expression (48) and broad-spectrum chemotherapies that differentially affect tumor heterogeneity (49). Given this complexity, identifying novel molecular targets for therapeutic and diagnostic applications based on genomic or transcriptional alternations in neuroblastoma is daunting. Our work suggests a different approach that is independent of genetic heterogeneity and upstream of the transcriptional networks that act as cell fate switches. Targeting the CCT protein-folding chaperonins for inhibition directly focuses therapeutics on the machinery that enables the cancer phenotype to emerge from genetic alterations and transcriptional programs. While associations between CCT2 and MYCN are not known, *CCT2* may be linked to overexpression of proto-oncogenes *CDK4* and *MDM2* as well as *CCND1* (8) and directly associates with *MYC* (8, 50). MYC overexpression is seen in a number of high-risk neuroblastoma patients (~10%) without *MYCN* amplification (51). In addition, substrates of CCT include drivers of aggressive neuroblastoma such as components of the RAS-MAPK and p53 signaling pathways (13, 15). Using SK-N-AS and IMR-32 cells, which are *MYCN* non-amplified or amplified, respectively, as well as being classified as MES versus ADRN states, we have shown that exogenous expression or depletion of CCT2 could impact cell size, adherence, migration, and viability through modulation of chaperonin’s protein-folding activity on actin and tubulin that was independent of genomic or transcriptomic differences in these cell lines. These data indicate that CCT2 could act as a molecular marker to successfully target those cells that acquire an invasive migratory phenotype, which is a hallmark of metastatic cancer, without the need to first identify genetic markers or transcriptional programs that, to date, are not fully characterized in neuroblastoma.

Protein-folding as a therapeutic target for cancer is supported by more than a decade of work with inhibitors against the molecular chaperone, HSP90. HSP90 proteins are highly conserved and ubiquitously expressed throughout most living systems. While there are five HSP90 family members, HSP90α and the constitutive form HSP90β are the primary mammalian isoforms, and their expression is induced by stress conditions. HSP90 chaperones act like biological catalysts that directly bind to their protein clients or substrates through non-covalent interactions. Interest in developing inhibitors of HSP90 emerged as a result of evidence that HSP90 could be detected on the cell surface and in the membranes of neuroblastoma cells (52). The consequence of HSP90 inhibition on neuroblastoma cells, both IMR-32 and SK-N-AS, was growth suppression and a decrease in MYC or MYCN expression. Increases in favorable neuroblastoma genes, *EFNB2, MIZ-1*, and *TrkA*, were also observed. However, increases in p53 resulted as well (53). Such studies led to the development and testing of novel HSP90 inhibitors in pediatric cancers. For example, SNX-2112, an oral HSP90 inhibitor, showed cytostatic effects at low doses against a panel of pediatric cancer cell lines and reduction of AKT and C-Raf over time (54). Other HSP90 inhibitors, including natural products like geldanamycin (GA), further demonstrated the *in vitro* and *in vivo* anti-cancer effects of HSP90 inhibition. However, the clinical use of such inhibitors is not approved by the FDA due to the structural instability of the drug as well as possible hepatotoxicity (55). Other HSP90 inhibitors tested include modifications of GA, producing 17-AAG that was used in phase I/II clinical trials in cancer patients and showed good tolerance. In pediatric patients with solid tumors, a phase I study of 17-AAG resulted in few DLTs (dose-limiting toxicities), and while no objective responses were noted, 3 out of 17 patients remained on therapy with stable disease (56). In another phase I trial of 17-AAG with fifteen pediatric patients, similar responses were reported (57). Though generally well-tolerated, subsequent adult clinical trials of HSP90 inhibitors resulted in short-lived outcomes, and resistance developed through activation of survival signaling pathways like JAK/STAT, YAP/TAZ, and TGF-beta induced EMT, or activation of HSF1 and the cytoprotective heat shock response (12, 58). HSP90 inhibitors in combination with other inhibitors such as STAT3 (58) or the simultaneous inactivation of multiple HSP90 family members like Grp94 and TRAP1 (22) were tested to improve outcomes. However, DLTs associated with achieving effective depletion of HSP90, combined with the complexity of attaining therapeutic dosing of drug combinations, may limit the use of HSP90 inhibition therapies in childhood cancers like neuroblastoma.

Positive aspects of pre-clinical and clinical trials with HSP90 inhibitors support the development of CCT as a therapeutic target. Three CCT inhibitors are reported in the literature. Two of these targets a specific interaction between CCT2 or CCT4 and tubulin or STAT3, respectively (59, 60). The compound I-Trp is an iodomethyl ketone warhead that alkylates Cys^354^ of β-tubulin and as a result disrupts the protein-protein interactions between the CCT2 and tubulin (61, 62). Anticarin-β is a natural coumarin compound from *Antiaris toxicaria* Lesch that prevents the maturation of STAT3 mediated by CCT4. This compound was first reported as a STAT3 inhibitor (63). The third CCT inhibitor, CT20p, was developed by our lab and is the first-in-kind to be substrate-independent (34) and could potentially function by blocking sites for nucleotide binding or inter- intra-ring contacts. Using CT20p encapsulated in polymeric NP, we achieved tumor regression in breast cancer and prostate cancer animal models as well as small cell lung cancer (SCLC) cell lines (26, 34, 38, 40). Using CT20-NPs, we showed that IMR-32 neuroblastoma cells are sensitive to the peptide’s cytotoxic effects. While CT20p has not yet been tested in clinical trials, we anticipate that cytoprotective pro-tumor effects associated with HSP90 inhibitors would not transpire given that reductions in CCT levels do not induce a heat shock response (17) and *cct* genes have fewer heat shock elements for binding heat shock transcription factors (HSF1 and HSF2) than comparable HSPs (6).

The clinical targeting of CCT could also extend to use as an indicator of disease progression for diagnostic purposes. CCT2 could be especially useful by liquid biopsy in the detection of cell-free CCT2 nucleic acids or CCT2-expressing CTCs. Unlike cell-free DNA or RNA, CTCs are short-lived tumor cells shed from primary or metastatic sites that contain comprehensive information on tumor status. CTCs thus are dynamic markers whose presence in blood could provide actionable information to guide neuroblastoma patient management (64). Using CCT2 as a marker for CTCs adds pertinent information on the potential for the detected CTCs to originate from an aggressive or invasive cancer. Previous studies to detect CTCs from neuroblastoma patients used immunological methods with antibodies targeting neuron markers like GD2, CD56, or CD90 (65–68). However, such methods, while specific, in the absence of an enrichment step, have low sensitivity. A different approach tested similar markers with the DepArray system, which uses dielectrophoresis to trap fluorescently labeled cells (69). While successful in pre-clinical studies, these methods relied upon the detection of a specific neuronal marker which may overlook cells that lack these markers. Detection of CCT, and specifically CCT2, is independent of cancer type or lineage markers and is based upon a biological function that is upregulated in cancers, especially those with invasive features. Others have used CCT subunits to predict disease outcomes which helped stratify breast cancer patients for treatment (70) or utilized as a liquid biopsy marker to monitor the progress of glioma patients (71), and even acted as a serum liver cancer biomarker in colorectal cancers (27). We found that CCT2 could be used to identify breast and small cell lung cancer (SCLC) cells spiked in the blood and further enhanced the detection of CTCs in the blood of SCLC patients (32). In total, our work with CCT in neuroblastoma reveals for the first time that protein-folding can be effectively inhibited by targeting a chaperonin. These data present a novel path toward developing new therapeutic and diagnostic approaches based on CCT2 that could improve the detection of neuroblastoma in patients and provide treatment options to limit both short-term and long-term side effects.

## Data availability statement

The original contributions presented in the study are included in the article/**Supplementary Material**. Further inquiries can be directed to the corresponding author.

## Author contributions

The manuscript was written with the contributions of all authors. AC, DN, OC, EL, ASK, JM, RB, TW, and ARK. AC, DN, OC, EL, JM, and RB contributed to data acquisition. EL and ASK contributed to the data analysis. AC, JM, TW, and ARK contributed to the experimental design and manuscript preparation. All authors have approved the final version of the manuscript.

## Funding

Funding was provided by the Live Like Bella Foundation (#21L11).

## Conflict of interest

The authors declare that ARK is a shareholder in Seva Therapeutics, Inc.

The remaining authors declare that the research was conducted in the absence of any commercial or financial relationships that could be construed as a potential conflict of interest.

## Publisher’s note

All claims expressed in this article are solely those of the authors and do not necessarily represent those of their affiliated organizations, or those of the publisher, the editors and the reviewers. Any product that may be evaluated in this article, or claim that may be made by its manufacturer, is not guaranteed or endorsed by the publisher.
