# Supplementary material for: Chaperonin containing TCP-1 (CCT/TRiC) is a novel therapeutic and diagnostic target for neuroblastoma
Source: Front Oncol. 2022 Sep 15;12:975088. doi: 10.3389/fonc.2022.975088 (PMC9520665; doi:10.3389/fonc.2022.975088)
Supplement: Supplementary file 1 [file DataSheet_1.pdf]

Chaperonin containing TCP-1 (CCT/TRiC) is a novel therapeutic and diagnostic target for neuroblastoma

Amanda Cox[1], Daniel Nierenberg[1], Oscar Camargo[1], Eunkyung Lee[2], Amr S. Khaled[3], Joseph Mazar[4], Rebecca J. Boohaker[5], Tamarah J. Westmoreland[4], and Annette R Khaled[1]

[1]Burnett School of Biomedical Science, College of Medicine, University of Central Florida, Orlando, FL, United States of America.

[2]College of Health Professions and Sciences, University of Central Florida, Orlando, FL, United States of America.

[3]Pathology and Laboratory Medicine, Orlando VA Medical Center, Orlando, FL, United States of America.

[4]Nemours Children's Hospital, Orlando, FL, United States of America

[5]Southern Research, Birmingham, AL, United States of America

Supplemental Files:

Table S1: RT-qPCR Primers

GAPDH	Forward: GAA GGT GAA GGT CGG AGT CAA C
	Reverse: TGG AAG ATG GTG ATG GGA TTT C
CCT3	Forward: TCAGTCGGTGGTCATCTTTGG
	Reverse: CCTCCAGGTATCTTTTCCACTCT
CCT2-FLAG	Forward: CAG AGG TGA TTC TGC GTG TG
	Reverse: TGT CGT CGT CGT CCT TGT AG
CCT2	Forward: GTT GGA GAG AAG CCA CGA AG
	Reverse: GTT GCC AGA GCC TTT CAG TC

Table S2: CCT2 Sequences

Sequence:
MASLSLAPVNIFKAGADEERAETARLTSFIGAIAIGDLVKSTLGPKGMDKILLSSGRDASLMVTN
DGATILKNIGVDNPAAKVLVDMSRVQDDEVGDGTTSVTVLAAELLREAESLIAKKIHPQTIIAGW
REATKAAREALLSSAVDHGSDEVKFRQDLMNIAGTTLSSKLLTHHKDHFTKLAVEAVLRLKGSG
NLEAIHIIKKLGGSLADSYLDEGFLLDKKIGVNQPKRIENAKILIANTGMDTDKIKIFGSRVRVDST
AKVAEIEHAEKEKMKEKVERILKHGINCFINRQLIYNYPEQLFGAAGVMAIEHADFAGVERLALV
TGGEIASTFDHPELVKLGSCKLIEEVMIGEDKLIHFSGVALGEACTIVLRGATQQILDEAERSLHD
ALCVLAQTVKDSRTVYGGGCSEMLMAHAVTQLANRTPGKEAVAMESYAKALRMLPTIIADNA
GYDSADLVAQLRAAHSEGNTTAGLDMREGTIGDMAILGITESFQVKRQVLLSAAEAAEVILRVD
NIIKAAPRKRVPDHHPGGGGCENLYFQS

Sequence:
CATATGGCGAGCCTGAGCCTGGCGCCGGTGAACATCTTCAAAGCGGGTGCGGACGAGGAGC
GTGCGGAAACCGCGCGTCTGACCAGCTTCATCGGCGCGATCGCGATTGGTGACCTGGTGAAG
AGCACCCTGGGCCCGAAGGGTATGGATAAAATTCTGCTGAGCAGCGGTCGTGATGCGAGCC
TGATGGTGACCAACGATGGCGCGACCATCCTGAAGAACATTGGTGTTGACAACCCGGCGGC
GAAAGTGCTGGTTGATATGAGCCGTGTGCAGGACGATGAGGTTGGCGACGGTACCACCAGC
GTGACCGTTCTGGCGGCGGAACTGCTGCGTGAGGCGGAAAGCCTGATCGCGAAGAAAATTC
ACCCGCAAACCATCATTGCGGGTTGGCGTGAAGCGACCAAAGCGGCGCGTGAAGCGCTGCT
GAGCAGCGCGGTGGACCATGGTAGCGATGAGGTTAAGTTCCGTCAGGACCTGATGAACATC
GCGGGTACCACCCTGAGCAGCAAACTGCTGACCCACCACAAGGATCACTTTACCAAACTGG
CGGTGGAGGCGGTTCTGCGTCTGAAAGGCAGCGGTAACCTGGAAGCGATTCACATCATTAA
GAAACTGGGTGGCAGCCTGGCGGACAGCTACCTGGATGAGGGCTTCCTGCTGGACAAGAAA
ATCGGTGTGAACCAACCGAAGCGTATTGAAAACGCGAAAATCCTGATTGCGAACACCGGCA
TGGACACCGATAAGATCAAAATTTTTGGTAGCCGTGTGCGTGTTGATAGCACCGCGAAGGTG
GCGGAGATCGAACACGCGGAGAAGGAAAAAATGAAGGAGAAAGTTGAACGTATCCTGAAA
CACGGCATCAACTGCTTCATTAACCGTCAGCTGATTTACAACTATCCGGAACAACTGTTCGG
CGCGGCGGGTGTTATGGCGATCGAGCACGCGGACTTTGCGGGTGTGGAACGTTGGCGCTGGT
TACCGGTGGCGAGATTGCGAGCACCTTCGATCACCCGGAACTGGTGAAGCTGGGCAGCTGC
AAACTGATCGAGGAAGTTATGATTGGCGAGGACAAGCTGATCCACTTTAGCGGCGTGGCGC
TGGGTGAAGCGTGCACCATCGTTCTGCGTGGTGCGACCCAGCAAATTCTGGACGAGGCGGA
ACGTAGCCTGCATGATGCGCTGTGCGTGCTGGCGCAGACCGTTAAAGATAGCCGTACCGTGT
ATGGTGGCGGTTGCAGCGAGATGCTGATGGCGCATGCGGTGACCCAACTGGCGAACCGTAC
CCCGGGCAAAGAGGCGGTTGCGATGGAAAGCTACGCGAAAGCGCTGCGTATGCTGCCGACC
ATCATTGCGGACAACGCGGGTTATGACAGCGCGGATCTGGTTGCGCAACTGCGTGCGGCGC
ACAGCGAGGGTAACACCACCGCGGGTCTGGACATGCGTGAAGGCACCATCGGTGATATGGC
GATCCTGGGTATTACCGAGAGCTTCCAGGTGAAGCGTCAAGTTCTGCTGAGCGCGGCGGAG

GCGGCGGAAGTGATTCTGCGTGTTGATAACATCATTAAAGCGGCGCCGCGTAAGCGTGTTCC
GGACCACCACCCGGGCGGCGGCGGCTGCGAAAATCTGTACTTCCAAAGCGGGATCCGGAAT
TC
AGADEERAETARLSSFIGAIAIGDLVKSTLGPKGMDKILLSSGRDASLMVTNDGATILKNIGVDNP
AAKVLVDMSRVQDDEVGDGTTSVTVLAAELLREAESLIAKKIHPQTIIAGWREATKAARQALLN
SAVDHGSDEVKFRQDLMNIAGTTLSSKLLTHHKDHFTKLAVEAVLRLKGSGNLEAIHVIKKLGG
SLADSYLDEGFLLDKKIGVNQPKRIENAKILIANTGMDTDKIKIFGSRVRVDSTAKVAEIEHAEKE
KMKEKVERILKHGINCFINRQLIYNYPEQLFGAAGVMAIEHADFVGVERLALVTGGEIASTFDHP
ELVKLGSCKLIEEVMIGEDKLIHFSGVALGEACTIVLRGATQQILDEAERSLHDALCVLAQTVKD
SRTVYGGGCSEMLMAHAVTQLASRTPGKEAVAMESYAKALRMLPTIIADNAGYDSADLVAQLR
AAHSEGKTTAGLDMKEGTIGDMSVLGITESFQVKRQVLLSAAEAAEVILRVDNIIKAAPR

Table S3. Number of tumor tissue types

**Figure 1A**	**# of tissue samples**
GTEx	7862
TCGA	734
TARGET	10535
**Figure 1B and 1E**	
Solid Tissue Normal	11
Primary Solid Tumor	286
**Figure 1C**	
Ganglioneuroblastoma	3
Non-germinomatous germ cell tumor	1
Hemangioblastoma	3
Teratoma	9
Neurofibroma/Plexiform	21
Oligodendroglioma	2
Malignant peripheral nerve sheath tumor (MPNST)	4
Langerhans Cell histiocytosis	4
Glial-neuronal tumor NOS	5
Sarcoma	5
Chordoma	6
Not Reported	2
Dysplasia/Gliosis	15
Meningioma	29
Other	34
Schwannoma	17
Cavernoma	1
Dysembryoplastic neuroepithelial tumor (DNET)	25
Ganglioglioma	49
Germinoma	5
Gliomatosis Cerebri	2
Craniopharyngioma	36
Brainstem glioma- Diffuse intrinsic pontine glioma	14

Ependymoma	93
Low-grade glioma/astrocytoma (WHO grade I/II)	254
Adenoma	3
Metastatic secondary tumors	7
Neurocytoma	3
Neuroblastoma	5
Atypical Teratoid Rhabdoid Tumor (ATRT)	30
Ewing's Sarcoma	8
High-grade glioma/astrocytoma (WHO grade III/IV)	103
Medulloblastoma	119
Pineoblastoma	3
Choroid plexus papilloma	15
Subependymal Giant Cell Astrocytoma (SEGA)	3
Supratentorial or Spinal Cord PNET	16
Choroid plexus carcinoma	4
Primary CNS lymphoma	1
Rhabdomyosarcoma	2
**Figure 1D**	
Acute Myeloid Leukemia, Induction Failure Subproject	32
ALL	194
AML	196
Clear cell sarcoma of Kidney	13
Neuroblastoma	162
Wilms Tumor	126

Table S4. Percent of neuroblastoma tumors with CCT2 score (NB641c, US Biomax)

CCT2 Score	% CCT2 Score
**0**	13%
**1**	5.6%
**2**	11.1%
**3**	53.7%
**4**	13%
**NR**	3.7%

NR, not readable

Table S5. CCT2 score for Neuroblastoma tissue microarray (TMA), NB641c (US Biomax), with with INSS stage and IHC marker (CD56/CgA)

Number	CD56	CgA	INSS stage	CCT2 score
45	-	-	I	0
46	-	-	I	0
1	+	-	I	0
2	+	-	I	1
3	++	++	I	0
4	++	++	I	0
39	++	+	I	0
40	++	+	I	0
43	++	-	I	1
44	++	-	I	1
32	++	++	I	2
31	++	++	I	3
9	++	-	I	4
10	++	-	I	4
8	+++	++	I	2
15	+++	+++	I	2
17	+++	+++	I	2
49	+++	+++	I	2
50	+++	+++	I	2
6	+++	++	IV	3
7	+++	++	I	3
11	+++	+++	I	3
12	+++	+++	I	3
13	+++	+++	I	3
14	+++	+++	I	3
16	+++	+++	I	3
18	+++	+++	I	3
19	+++	+++	I	3
20	+++	+++	I	3
21	+++	+++	IIB	3
22	+++	+++	IIB	3
23	+++	+++	IV	3
24	+++	+++	IV	3
29	+++	+++	I	3
30	+++	+++	I	3
35	+++	+	I	3

36	+++	+	I	3
37	+++	+	I	3
38	+++	+	I	3
41	+++	+++	I	3
42	+++	+++	I	3
47	+++	++	I	3
48	+++	++	I	3
51	+++	-	I	3
52	+++	-	I	3
53	+++	+	I	3
54	+++	+	I	3
5	+++	++	IV	4
25	+++	++	I	4
26	+++	++	I	4
33	+++	++	I	4
34	+++	++	I	4

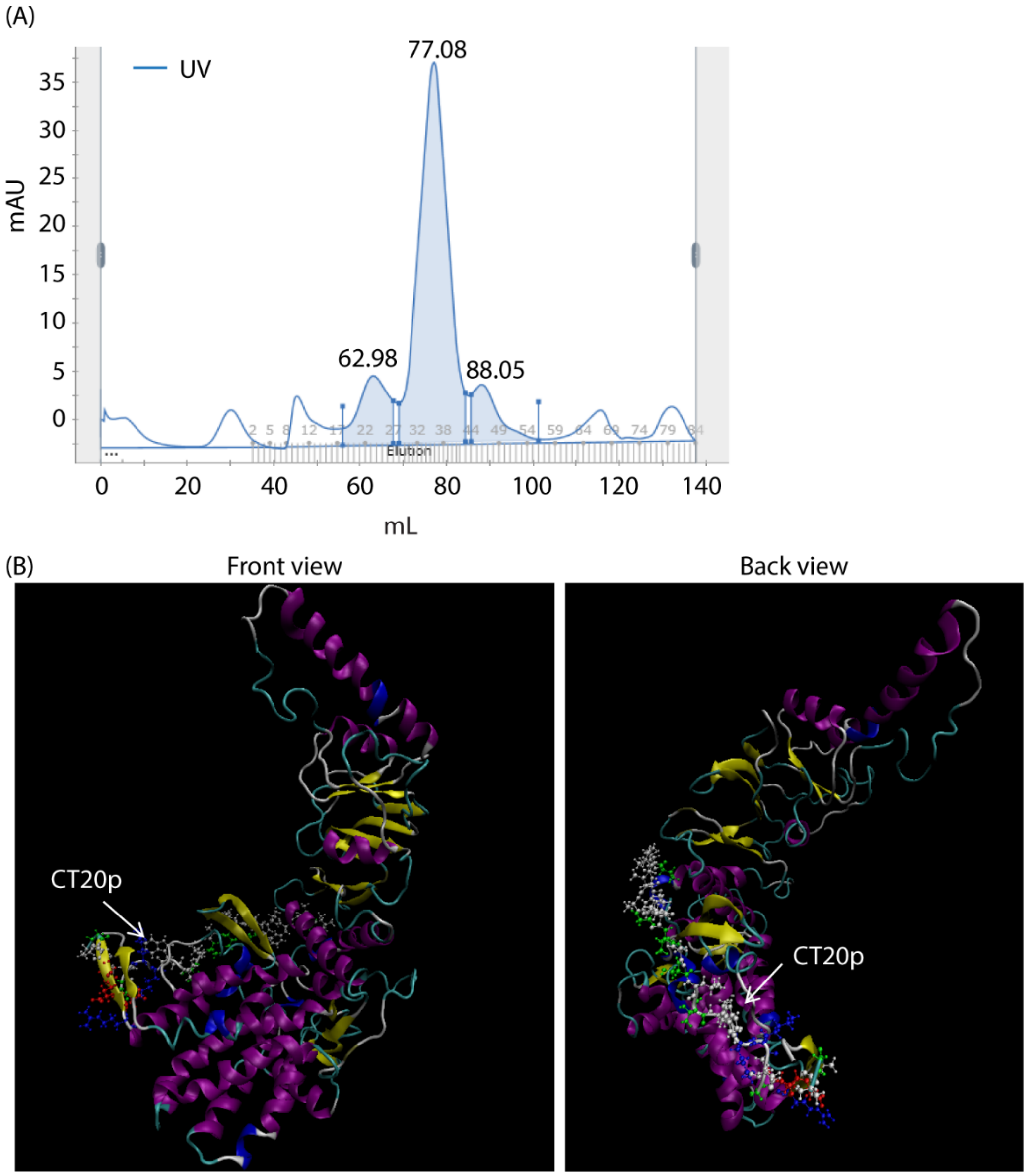

Figure S1. HiLoad 16 600 S200 CCTb60mM. (A) Purified protein from SEC column shows a single peak. (B) Homology modeling using MOE was done. Simulations of CT20p docking were performed with the atomic model of bovine CCT2 subunit derived from a 4.0 Angstrom cryo-EM map. Binding energies of CT20p on CCT2 are shown and most energetically feasible site for CT20p binding on CCT2 is shown by arrows.

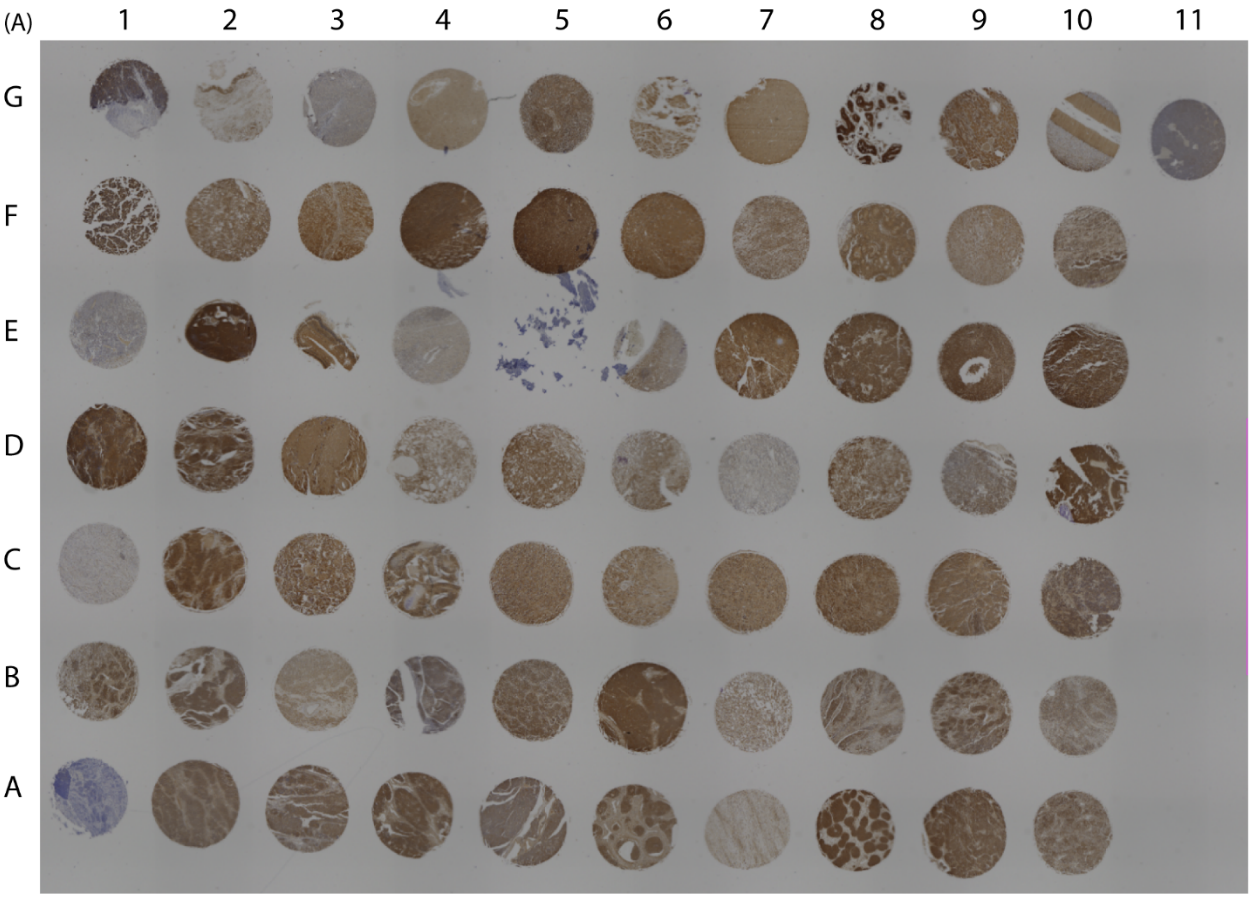

(B)

PC701

	1	2	3	4	5	6	7	8	9	10	11
G	Str	Med	Smo	Live	Lymp	Inf	Cer	Tes	Kid	Cer	Adr
F	Cer	Cer	Cer	Lym	Lym	Kid	Scr	Cer	Ret	Ret	
E	Eye	Eye	Eye	Liv	Liv	Liv	Cer	Cer	Ret	Lym	
D	Med	Med	Med	Tes	Tes	Tes	Tes	Tes	Tes	Eye	
C	Kid	Ret	Ret	Ret	Ret	Ret	Ret	Ret	Ret	Adr	
B	Kid	Kid	Kid	Kid	Kid	Kid	Kid	Kid	Kid	Kid	
A	Kid	Kid	Kid	Kid	Kid	Kid	Kid	Kid	Kid	Kid	

Legend

Adr	Adrenal Gland	Med	Mediastinum
Cer	Cerebellum	Ret	Retroperitoneum
Eye	Eye	Scr	Scrotum
Inf	Inferior Mediastinum/Diaphragm	Smo	Smooth muscle
Kid	Kidney	Str	Striated muscle
Liv	Liver	Tes	Testis
Lym	Lymph node	Cancerous Tissue	Normal Tissue

Figure S2. Pediatric malignant tumor tissue microarray (TMA) with normal tissue as control, PC701 (US Biomax), processed by IHC for CCT2. (A) Full image of TMA for stained for CCT2. (B) TMA map (n=70), duplicate cores.

(A)

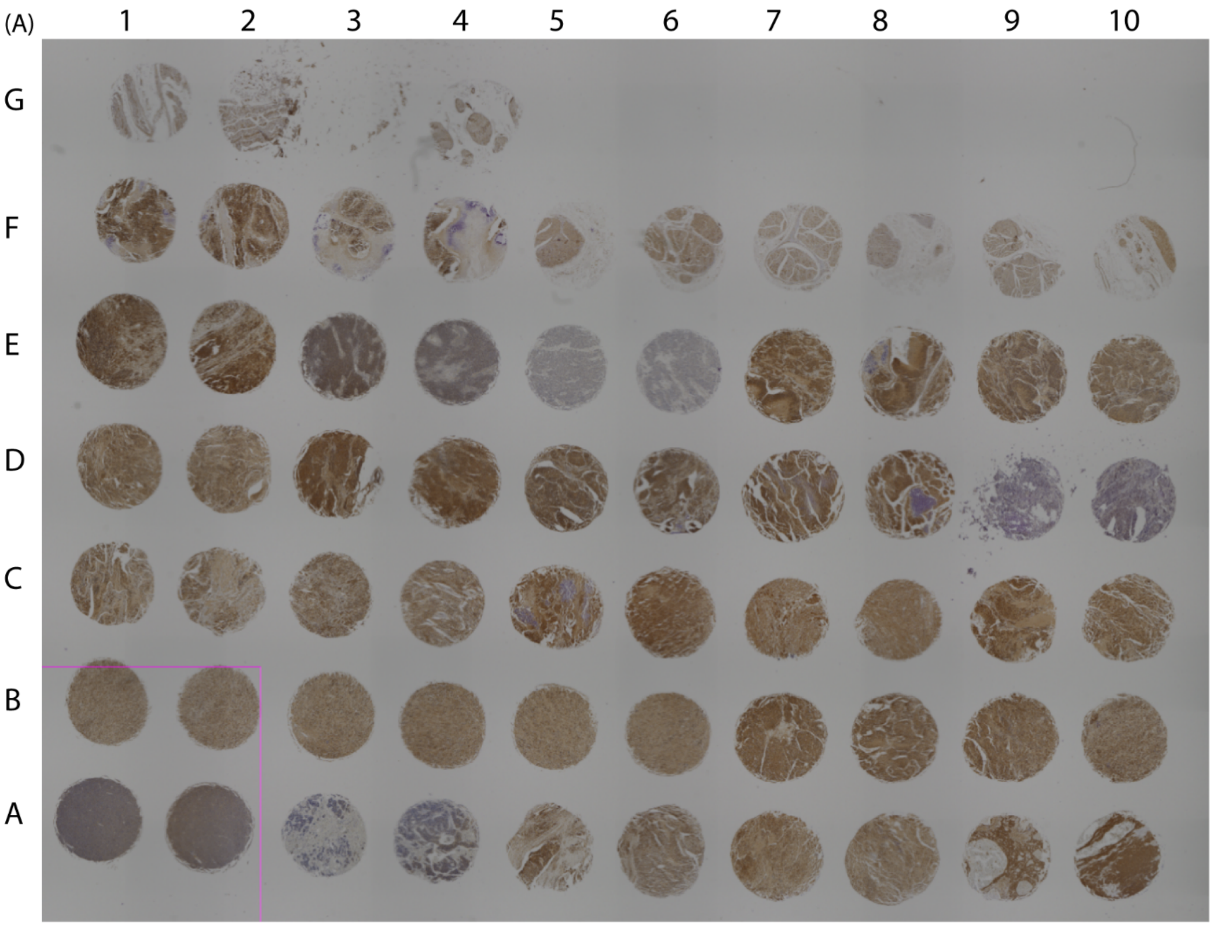

(B)

NB642c										
	1	2	3	4	5	6	7	8	9	10
G	Per	Per	Per	Per						
F	Adr	Adr	Adr	Adr	Per	Per	Per	Per	Per	Per
E	Med	Med	Pel	Pel	Pel	Pel	Adr	Adr	Adr	Adr
D	Ret	Ret	Adr	Adr	Med	Med	Med	Med	Med	Med
C	Ret	Ret	Ret	Ret	Ret	Ret	Ret	Ret	Ret	Ret
B	Ret	Ret	Ret	Ret	Ret	Ret	Ret	Ret	Ret	Ret
A	Ret	Ret	Ret	Ret	Ret	Ret	Ret	Ret	Ret	Ret
Legend										
	Adr	Adrenal Gland			Med	Mediastinum/left posterior				
	Pel	Pelvic Cavity			Ret	Retroperitoneum				
	Per	Peripheral nerve			Cancerous Tissue		Normal Tissue			

Figure S3. Neuroblastoma and peripheral nerve tissue microarray (TMA), with INSS stage, IHC marker (CD56/CgA), NB641c (US Biomax), processed by IHC for CCT2. (A) Full image of TMA stained for CCT2. (B) TMA map. (n=27), duplicate cores. Legend: Adr- Adrenal gland, Med- Mediastinum/left posterior , Pel- Pelvic cavity, Per- Peripheral nerve, Ret- Retroperitoneum.

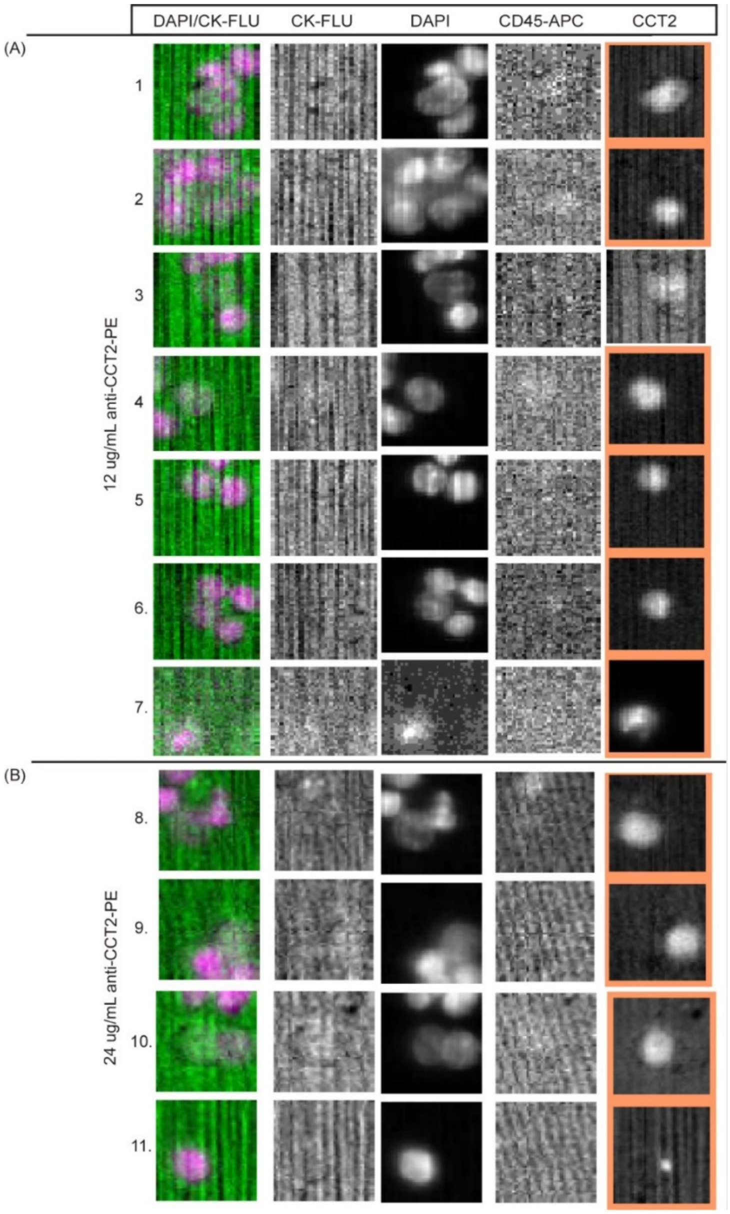

Figure S4. Representative images from Analyzer II for manually stained IMR-32 cells. CCT2-PE antibody concentration was 12 ug/mL (A) and 24 ug/mL (B). Column one is overlay of columns 2 and 3 which are cytokeratin and DAPI respectively, column 4 is CD45 marker for leukocytes and column 5 is for CCT2-PE antibody.
